# Outcomes of a penicillin allergy de-labelling implementation intervention in a UK hospital: a prospective, single centre, type 2 hybrid effectiveness-implementation study evaluated using mixed methods

**DOI:** 10.1186/s43058-026-01008-8

**Published:** 2026-06-11

**Authors:** Neil Powell, Daniel Hearsey, Robert West, Mathew Upton, Bridie Kent, Sarah Tonkin-Crine, Jonathan Sandoe

**Affiliations:** 1 Pharmacy Department Royal Cornwall Hospital Trust, Truro, Cornwall, UK; 2 Leeds Institute of Health Sciences, University of Leeds, Leeds, UK; 3 School of Biomedical Sciences, University of Plymouth, Plymouth, UK; 4 School of Nursing and Midwifery, University of Plymouth, Plymouth, UK; 5 Nuffield Department of Primary Care Health Sciences, University of Oxford, Oxford, UK; 6 Leeds Institute of Medical Research, University of Leeds, Leeds, UK

## Abstract

**Background:**

Non-allergy specialist healthcare workers removing low-risk penicillin allergy (penA) records, enabling patients to receive penicillin antibiotics, is safe but not common practice. We developed a behavioural change implementation intervention to support non-allergist healthcare workers to remove incorrect penA records from adult inpatients (penicillin allergy de-labelling; PADL). This study aimed to evaluate the effectiveness of the implementation intervention strategy and its impact on delabelling and antibiotic prescribing.

**Methods:**

The implementation intervention was launched 10^th^ June 2024. This prospective, single centre, type 2 hybrid effectiveness-implementation study was evaluated using mixed methods: process evaluation, qualitative and quantitative study designs. The proportion of patients delabelled was measured. Antibiotics were grouped into the three WHO AWaRe categories with differences in antibiotic use between the de-labelled and non-de-labelled measured. Pearson chi-squared tests, Welch’s t-tests or Wilcoxon matched-pairs signed-rank tests were used.

**Results:**

Between 10^th^ June and 13^th^ December 2024, the mean number of penicillin allergy records removed from the inpatient electronic prescribing system increased from 18.2/month pre-intervention to 42.7/month post intervention. Of 186 patients de-labelled with full data capture, the majority 126 (67.7%) were de-labelled by the antimicrobial pharmacists and the remainder by ward doctors or ward pharmacists across 21 clinical specialties. Antibiotic exposure, when grouped by WHO AwARe category, was not significantly different between de-labelled and not de-labelled groups. Of 138 patients with a penA record in their primary care records, there was evidence of communication with the GP to amend the primary care penA record for 94 (68.1%) patients of which 42 (44.7%) were actioned by GP surgeries. The PADL intervention was accepted by most patients, who considered hospital a safe place to be tested. Awareness of the PADL intervention was generally high across all healthcare workers. The PADL process was acceptable to all interviewed healthcare workers and aligned with staff roles and hospital processes but competing priorities were commonly cited as a barrier.

**Conclusions:**

The PADL implementation intervention successfully increased non-specialist workforce PADL of low-risk patients. There is opportunity to further optimise PADL but competing priorities are a challenge. We found non-statistically significant impact of PADL on antibiotic use.

**Supplementary Information:**

The online version contains supplementary material available at 10.1186/s43058-026-01008-8.

Contributions to the literature

- We describe an increase in penicillin allergy de-labelling following introduction of a behaviour change implementation intervention led by antimicrobial pharmacists and delivered by a multidisciplinary healthcare workforce.
- We have demonstrated pharmacists can lead and champion penicillin allergy de-labelling interventions within hospitals and incorporate it as part of the already well-established medicines reconciliation process.
- Contrary to other studies we did not see an antimicrobial stewardship benefit. However, the sensitivity of the analysis may have been affected by the metric used to for antibiotic prescribing, short follow-up period and differences between the de-labelled and not delabelled populations.

## Introduction

Fifteen percent of hospitalised patients report a penicillin allergy (penA), which prevents use of first line penicillin-based antibiotics, instead they receive alternative antibiotics which are associated with a greater risk of adverse events, higher acquisition costs and antimicrobial resistance [1, 2] Over ninety percent of patients with a penA record are able to tolerate penicillin after formal allergy testing and therefore many of these associated harms are potentially avoidable [3]. The WHO’s AWaRe classification categorises antibiotics into one of three groups, Access, Watch and Reserve, and promotes use of antibiotics in the Access group over those in the other two groups for reasons of antimicrobial resistance and safety [4]. In a previous study, we found inpatients with penA records were four times more likely to receive antibiotics from the Watch and Reserve groups than those without a penA record [5].

Traditionally penA testing has been the role of allergy specialists, but their paucity and the large number of patients with penA, has prompted non-allergists to explore penicillin allergy assessment. Removal of labels from those deemed low risk for true allergy, is termed penicillin allergy de-labelling (PADL). Non-allergist delivered PADL is safe and has a positive impact on antimicrobial stewardship through increased penicillin antibiotic prescribing over second line agents [6, 7]. Removing incorrect penA records has been shown to increase penicillin prescribing in primary care patients and therefore PADL is expected to increase the use of Access group antibiotics in hospital, of which many are penicillin antibiotics, and reduce the use of non-Access group antibiotics such as carbapenems and quinolones and thereby reduce the associated harms associated with non-penicillin antibiotic prescribing [8, 9].

Non-allergist inpatient-delivered PADL is a complex intervention that requires a number of patient and healthcare worker behaviours to be targeted in order to facilitate its delivery [10]. We followed an integrated approach to develop a behavioural change implementation intervention that combined theory-based, evidence based and person-based approaches designed to support non-allergist healthcare workers to remove incorrect penA records from medical and surgical adult inpatients. [11–13]. Development of the implementation intervention has been described previously [14]. This study aimed to evaluate and report on both the implementation strategy and the effectiveness of the PADL intervention using outcomes informed by the Consolidated Framework for Implementation Research (CFIR) outcome addendum [15].

## Methods: description

### Design

A prospective, single centre, type 2 hybrid effectiveness-implementation study design, evaluated using mixed methods; process evaluation, qualitative and quantitative (quasi-experimental) study designs [16]. The CFIR outcome addendum was used to identify implementation intervention and PADL intervention outcomes [14, 15]. Acceptability, appropriateness and feasibility of PADL were explored via semi-structured interviews and are reported elsewhere [17–19].

The implementation intervention was adopted as routine clinical service by the study organisation and delivered by HCWs. We used the Standards for Reporting Implementation Studies (StaRI) Statement to report this study (see appendix) [20].

### Context

750 bed district general hospital in the Southwest of England with a mature antimicrobial stewardship programme. Awareness of PADL amongst HCWs is high due to previous PADL work in the study hospital prior to launch of this intervention with several publications in infection journals [2, 5, 21], and general medical journals [22, 23]. A three-month PADL pilot was undertaken between November 2022 and February 2023 [24]. This implementation intervention was launched 10th June 2024 on all acute adult medical and surgical wards.

### Description of the intervention

The PADL pathway is described elsewhere (appendix A). All grades of doctors, pharmacists, medicines optimisation pharmacy technicians (MOPTs) and nurses delivered all, or parts of the PADL intervention.

Patients categorised as ‘low risk’ of true PenA were offered direct de-labelling, (based on history alone (DDL), or a direct oral challenge test (DOCT). A process of ‘medicines reconciliation’, undertaken by ward pharmacists and MOPTs, is well established in UK hospitals [25]. We added penicillin allergy focused history questions and the risk assessment tool to the hospital pharmacy medication reconciliation desktop folder on personal computers used in ward areas. (appendix A). The penA focused questions, with the answers given by patients, were attached to the patient’s electronic prescribing records (WellSky EMR Systems) as a ‘pharmaceutical care plan note’ and given the title ‘PADL’. Doctors were encouraged to follow the same process as described for the ward pharmacy teams, particularly when penA records were preventing first line antibiotic treatment. Ward pharmacists and ward doctors were encouraged to work together to undertake the tasks described.

The Antimicrobial stewardship team reviewed local intranet page daily (Monday to Friday) to identify patients with PADL notes that needed action (risk assessment and, where appropriate, patients offered de-label).

### Description of the implementation strategy

The implementation strategy was delivered in three phases presented in detail elsewhere (summary in appendix A) [14].

## Methods: evaluation

### Implementation strategy

**Fig. 1:**
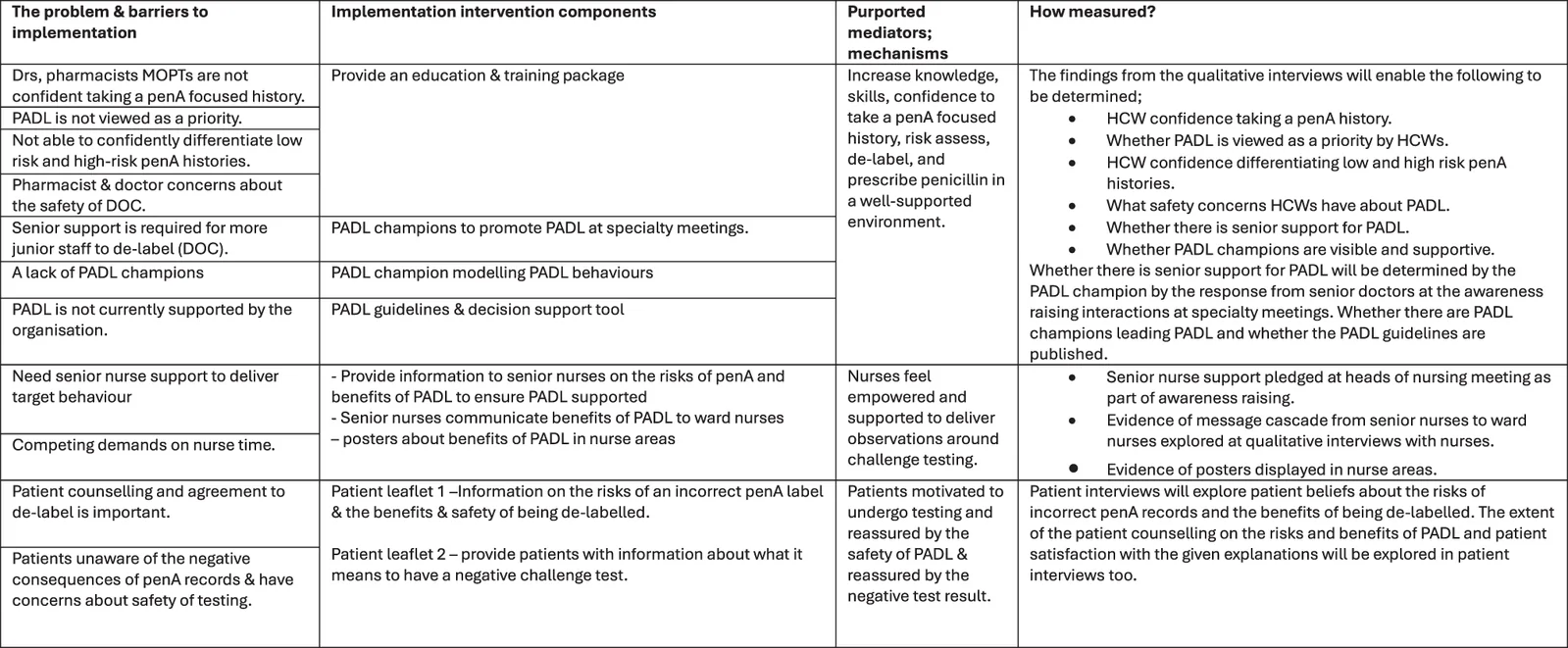
Shows the identified barriers implementing PADL, the implementation components developed to overcome those barriers, the purported mediators, using the theoretical domains framework, of those desired changes in HCW and patient behaviours and the process evaluation, their objectives and the measured outcomes in the process evaluation measures

The following implementation strategy outcomes were determined, guided by the CFIR, and shown in Table 1 [15]. Fig. 1 shows how the implementation intervention was expected to work and the process evaluation objectives and outcomes related to the mechanism by which the strategy was expected to work.

**Table 1:** Shows the implementation strategy outcomes and how they were measured

CFIR measured outcomes	How the CFIR outcomes were measured	The level(s) of the measured outcome (patient, provider, team, organisation, system).	Component outcome measures (Implementation, Intervention, Process).
Adoption	• Verbal approval and support for PADL at hospital level (MPC), medical and surgical specialties level, senior pharmacy level and senior nurse level. Level of support was recorded by the PADL champion while attending departmental meetings and verbal support from representatives from each specialty was documented (as “supported” or “not supported”).	• Organisation, teams, provider,	• Implementation
	• The breath of medical and surgical specialties engagement with PADL was gauged by the specialty caring for the patient at the time of de-labelling.	• Teams	• Implementation
	• Semi-structured interviews with patients and HCWs explored barriers and enablers to adoption and through these interviews how well the intervention was adopted was gauged.	• Provider and patient	• Implementation
	• Engagement with training modules by HCW groups was determined.	• Provider	• Implementation
Implementation	• Patients with a penA record were identified who had spent some or all their time on a surgical or medical ward and the number of these patients who had had their penA record removed each month from the electronic prescribing system. We did an interrupted time series analysis of monthly de-label rate between January 2021 and August 2025. There were five distinct phases during this period: pre-feasibility study, feasibility study, post-feasibility study, implementation intervention, and post-implementation intervention. Monthly de-labelling counts were modelled as Poisson-distributed variables, with the mean rate parameter varying according to the phase in which each month falls. Months were sequentially numbered from 1 to 56, with January 2021 designated as Month 1 and August 2025 as Month 56. Model fit and credibility were assessed through graphical diagnostics, including a scatter plot and stepwise trend overlays, to evaluate the adequacy of the segmented Poisson model.	• Provider and patient	• Implementation
	• In addition, we recorded: number of discharged patients each month with PADL notes added to EPMA during their inpatient stay; which HCW group took the penA history; which HCW group made the decision to de-label; and the number of different personnel within each HCW group engaging with each PADL step. To determine missed opportunity, the number of patients with penA record who were prescribed a non-penicillin antibiotic and without a PADL note was determined.	• Provider and patient	• Implementation
Acceptability, appropriateness and feasibility	• Semi-structured interviews were undertaken to explore acceptability, appropriateness and feasibility of the PADL pathway with patients who have been offered PADL and doctors, nurses and pharmacy staff working in adult inpatients surgical and medical ward areas and described elsewhere [18]	• Provider and patient	• Implementation
Intervention (PADL)	• Proportion of low-risk patients successfully de-labelled with reasons for not being de-labelled captured.	• Patient	• Innovation
	• The impact of PADL on both antibiotic exposure and antibiotic DDDs, grouped by the WHO AwARe categories, was explored using multi variable logistic regression analysis.	• Patient	• Innovation
	• All risk assessed patients underwent a medical notes review after being discharged from hospital to identify any antibiotic-associated harm after the PADL time point or, for non-de-labelled patients, past the point of penA risk assessment.	• Patient	• Innovation
Process evaluation	• The outcomes of the PADL champions awareness raising meetings with senior HCWs groups and the findings from the qualitative interviews with HCWs and patients were used to evaluate the implementation processes designed to embed the PADL intervention into clinical practice.	• Provider and patient	• Implementation and innovation

PADL=penicillin allergy de-labelling, HCW=healthcare worker, MPC=Medicines Practice Committee, CFIR=Consolidated Framework for Implementation Research

### Economic evaluation

To determine implementation strategy costs, staff resource required to deploy each component of the PADL implementation intervention were costed in British pounds using estimated staff time to complete tasks and using salary mid-points.

Intervention costs were determined using an estimation of the average time taken to complete each PADL step and healthcare profession salary mid-point (British Pounds).

### Sample size

To assess the statistical power of the study, an initial scenario was modelled in which the primary outcome, defined as the number of non-Access defined daily doses (DDDs) prescribed following intervention, was compared between two patient groups: those who underwent PADL and those who did not. Assuming a standard deviation of 10 DDDs, a sample size of 150 de-labelled patients and 300 non-de-labelled patients was estimated to provide 80% power to detect a mean difference of 2.8 DDDs between groups at a significance level of 0.05. This calculation was intended as a preliminary guide, recognising that the final analysis might require regression modelling to adjust for potential confounding variables and accommodate a skewed outcome distribution.

### Descriptive Analysis

To characterise the study population and explore patterns in antibiotic prescribing, cross-tabulations of patient demographic and clinical variables were presented by de-labelling status. Clinical variables included binary indicators of Access and non-Access antibiotic prescriptions, as well as DDDs prescribed before and after assessment.

Where appropriate, statistical tests were applied to guide interpretation of group differences: Pearson chi-squared tests for categorical variables, Welch’s t-tests for continuous variables assumed to be normally distributed (e.g., age), Wilcoxon matched-pairs signed-rank tests for continuous variables with non-normal distributions (e.g., DDDs). These tests were used to support descriptive comparisons and were not interpreted as formal hypothesis tests.

### Statistical analysis plan

The primary outcome variable was the number of non-Access DDDs prescribed per patient post-intervention. The principal exposure of interest was de-labelling status. Covariates considered for inclusion in the regression model included the number of Access and non-Access DDDs prescribed prior to intervention, and the treated organ system, which served as a proxy for clinical indication and antibiotic necessity.

Given the anticipated non-normal distribution of DDDs, a transformation of the outcome variable using log(0.1 + DDD) was considered, with the constant added to avoid undefined values for zero doses. This transformation aimed to approximate normality in the residuals of the regression model. Associations between de-labelling and the outcome, as well as other covariates such as age and sex, were to be assessed using Wald tests of the corresponding regression coefficients.

#### Post-hoc revisions to the analysis plan

Following data collection, several observations prompted a revision of the planned statistical approach:

1. The observed difference in mean non-Access DDDs between de-labelled and non-de-labelled patients was substantially smaller than the threshold estimated in the initial power calculation.
2. Baseline demographic and clinical characteristics between the two groups were largely comparable.

These findings suggested that regression modelling might not be necessary and could potentially introduce bias rather than mitigate it. Moreover, the limited observed effect size indicated that the study was underpowered to detect a statistically significant association, even with adjustment.

Consequently, the emphasis of the analysis shifted from formal statistical inference to descriptive reporting of the study findings.

### Process evaluation

Qualitative interviews with HCWs and patients were used to evaluate the implementation processes designed to embed the PADL intervention into clinical practice, and reported elsewhere [18, 19]. In brief, purposive sampling was used to identify medical doctors, surgeons, nurses, pharmacists and MOPTs with a range of years’ experience and patients representing the following groups [1]: suitable for direct oral challenge (DOC) or direct de-label (DDL) but declined testing [2], suitable for DOC and accepted testing [3], suitable for DDL and accepted de-label. Fifteen HCWs (six doctors, five pharmacists and four MOPTs but despite repeated emails and in person communication with nurse leaders, nurse participants were not identified) and twenty patients agreed to be interviewed. Inductive reflexive thematic analysis was used to analyse transcripts [26].

## Results

### Implementation strategy

**Fig. 2:**
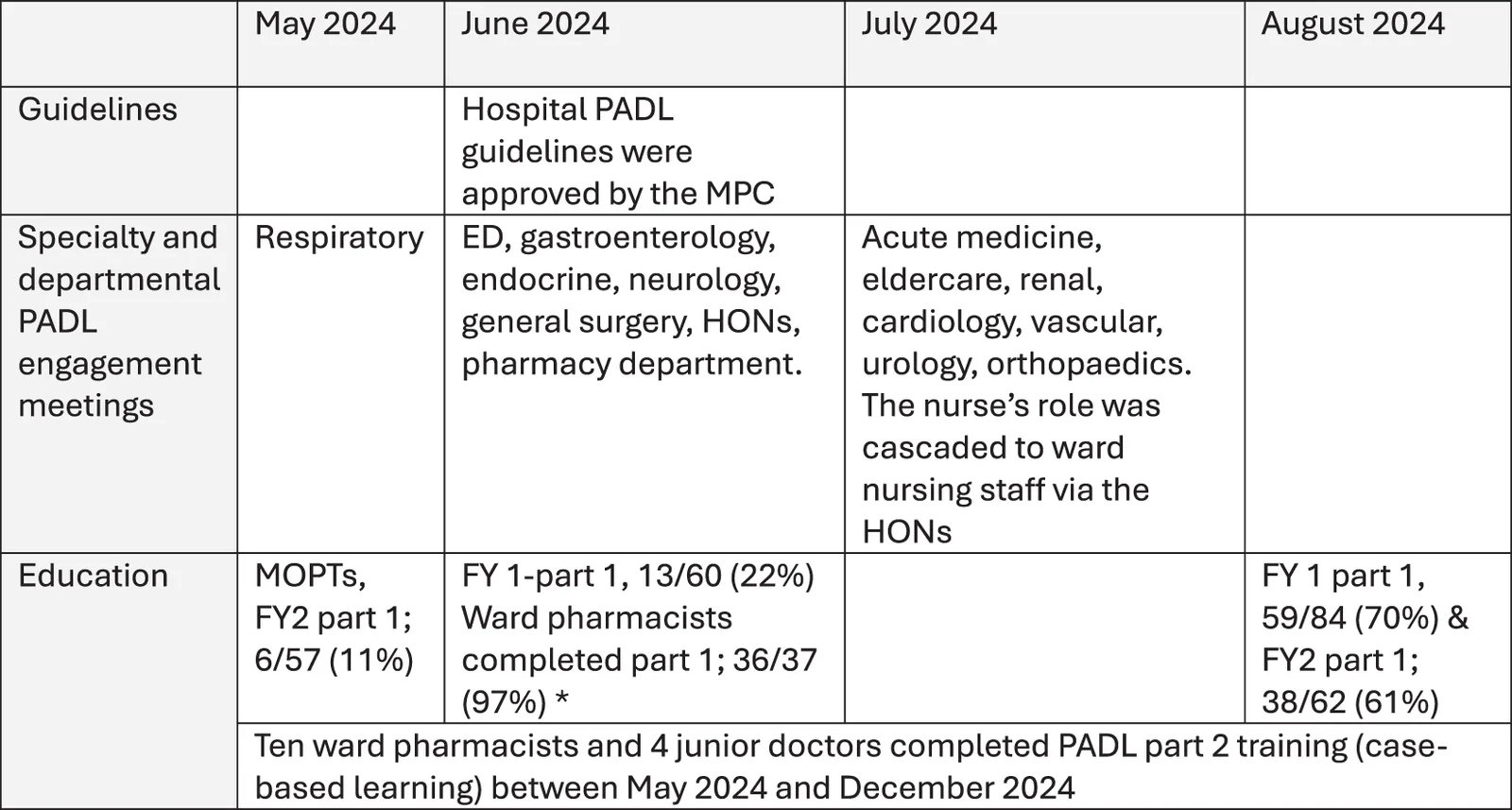
Specialty and departmental meetings attended by the PADL champion to both raise awareness and obtain support for PADL, all the teams were supportive of PADL with no concerns raised ENT and maxillofacial specialty meetings were not attended. *final pharmacist completed part 1 October 2024. Medicines practice Committee = MPC, emergency department = ED, head of nursing = HONs, foundation Years doctor 1 and 2 (FY1 and FY2), penicillin allergy de-labelling = PADL

Figure 2 shows the timeline for PADL engagement meetings with specialties and departments, guideline publication and education roll out.

### Removal of penA notes from EPMA

**Fig. 3:**
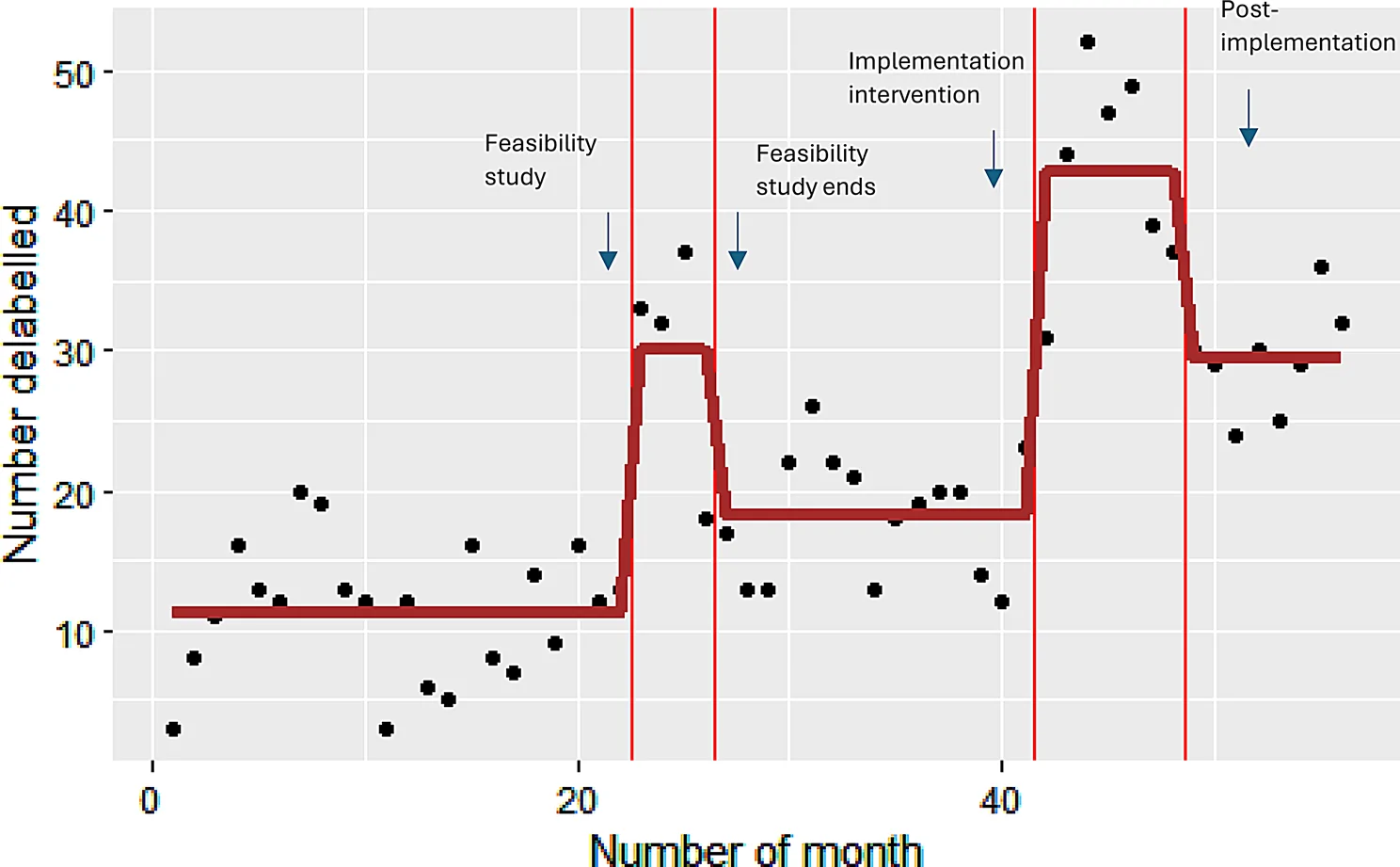
Presents a scatter plot overlaid with a step line graph, illustrating the temporal dynamics of patients delabelling across a series of monthly intervals. Individual data points are represented by dots, capturing the observed values for each month. Superimposed on these data points is a thick step line, which models the underlying trend or segmentation within the dataset. Additionally, four vertical lines are included in the figure, indicating change points for the observation period

The electronic prescribing system was used to identify patients with a removed penA record. All period-specific increases in mean de-labelling rates relative to the pre-feasibility baseline were statistically significant (*p* < 0.001). For instance, the post-intervention phase exhibited a substantial increase of 18.11 patients per month compared to the pre-feasibility phase (29.38–11.27), underscoring the sustained impact of the intervention, see Table 2 and Fig. 3.

**Table 2:** Mean number of penA records removed from EPMA per specified period

Period	Estimate	95% Confidence Interval
Pre-feasibility	11.27	(9.93, 12.73)
Feasibility (November 2022-Feburary 2023)	30.00	(4.95, 35.69)
Post-feasibility (Feb 2023 -May 2024)	18.20	(16.13, 20.45)
Implementation intervention (June 2024-Dec 2024)	42.71	(38.05, 47.74)
Post-intervention (January 2025 – August 2025)	29.38	(25.78, 33.29)

Of all patients admitted with a penA record during the implementation intervention period, 1115/2397 (47%) had a structured penA history documented in EPMA (PADL note) and 1283/2397 (53%) didn’t. (see graph appendix B1 and B2). Table 3 shows the numbers of different healthcare worker groups adding PADL notes to EPMA each month.

**Table 3:** Shows the number of different healthcare workers adding penicillin allergy de-label (PADL) notes to the electronic prescribing and medicines information (EPMA) system by month

Month	All HCW	Pharmacist or trainee pharmacists	MOPT or trainee techs	Doctors
June	21	14	7	
July	47	25	22	
August	54	31	23	
September	46	22	22	2 FY MM rotation
October	53	26	26	1 FY MM rotation
November	43	21	21	1 (ED junior)
December	43	20	21	1 (ED junior)
**Average**	**44**	**18**	**20**	

FY = foundation years doctor (years 1 and 2), MM = medical microbiology, ED = emergency department

### PenA patients prescribed any antibiotic

**Fig. 4:**
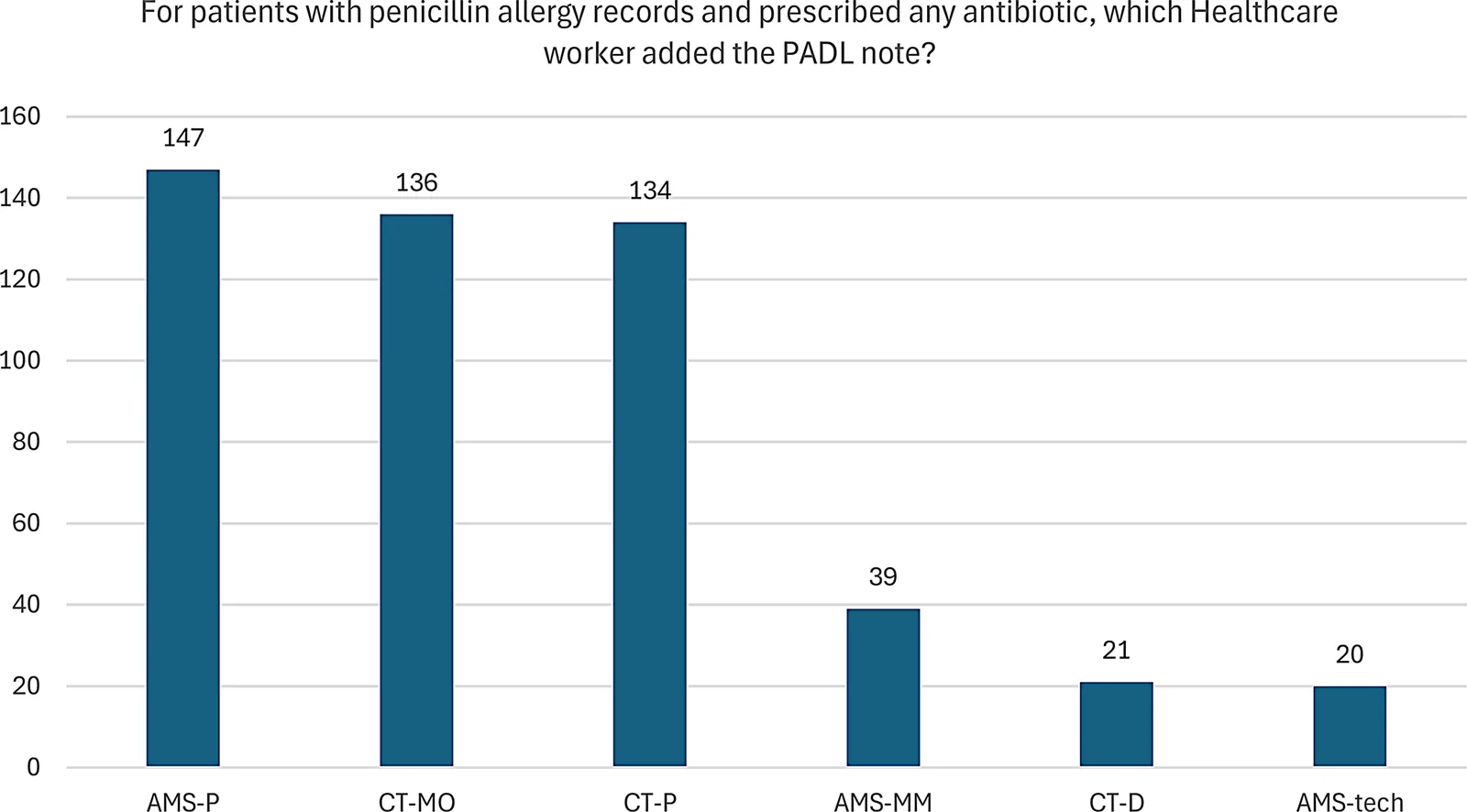
Shows which HCWs took the penA history. CT-D documented their penA history in the medical notes. All other groups added a PADL note. AMS-P antimicrobial stewardship pharmacist, CT-P clinic team pharmacist, CT-MO clinical team medicines optimisation pharmacy technician, CT-D clinical team doctor, AMS-tech antimicrobial stewardship technician

The mean proportion of penA patients on an antibiotic and with a PADL note was 60% (appendix graph B3). Of the 735 penA patients prescribed any antibiotic and who had a structured penA history documented on EPMA, 549 (74.7%) were risk assessed. After removing 52 patients who had opted out of their data being used for research, we report on 497 patients with PADL risk assessments. Of these, 291/497 (58.6%) of the penA histories were taken by the ward teams working in clinical areas and the AMS pharmacy team took 206/497 (41.4%) of the penA histories; (See Fig. 4).

Of the 186 patients de-labelled, 126 (67.7%) were de-labelled by AMS pharmacists (i.e. the two PADL champions), 31 (16.7%) clinical team doctors, 24 (12.9%) clinical team pharmacist, 4 (2.2%) AMS-MM and 1 (0.5%) by the MOPT (appendix B4).

Patients were de-labelled in 21 clinical specialties with the highest numbers de-labelled in eldercare 32 (17.2%), general surgery 30 (16.1%) and acute medicine 28 (15.1%). (appendix B5 & B6).

### Acceptability, appropriateness and feasibility (qualitative study outcomes)

The findings of these qualitative studies are reported in detail elsewhere, but they are summarised here [14, 18].

The PADL intervention was accepted by most patients, although some patients felt that during a period of acute illness was not the right time to be tested and they would have preferred to be assessed as an outpatient once discharged. Most patients reported receiving an adequate explanation of the PADL processes and the risks and benefits, although several patients did not recall being offered PADL, but acknowledged that they had several interventions discussed and the inpatient experience was overwhelming for some patients.

Awareness of the PADL intervention was high amongst junior (FY doctors) and consultants but low amongst the middle grades; training and awareness raising targeted these groups well but missed the middle grade doctors. The PADL process was acceptable to all interviewed HCWs and aligned with HCW roles and the Medicines Reconciliation process. PADL tools were more accessible to pharmacy teams than doctors due to the differences in how they access the PADL tools and their infrequency of use. Competing priorities were commonly cited as barriers to delivering PADL in ward areas, particularly by the medical staff who felt pharmacy staff were better positioned to deliver PADL.

### Intervention outcomes

497 patients were risk assessed over the study period; See table below for patient demographics (Table 4). The penicillin allergy focused history details reported by the 497 penA risk assessed patients are shown (appendix B7).

**Table 4:** Patient demographics. ‘Ethnicity other’ includes white Irish (2), white other (1), unknown (3), any other ethnic group (1), any other white/black (2), mixed white/Asian (1), any other white background (1)

Total number of patients	497		
Age mean (SD)	72 (SD 16.02)		
M:F ratio	190:307		
Ethnicity	White British 424 (85.3%); Cornish 62 (12.5%); other^*^ 11 (2.2%);		
Infection treated (n)	Respiratory Abdomen skin/soft tissue urinary Infection unknown source multiple infection sites nil infection nil antibiotic prescribed prophylaxis Bone & joint Infection site not documented Gynaecology neutropenic sepsis Central Nervous System Dental infection Endovascular mycobacterium intracellulare		144 115 78 77 20 14 9 8 8 7 6 4 4 1 1 1
Highest NEWS score on the day of de-label or penA assessment, if not de-labelled (median (IQR))	DOC DDL Not de-labelled	median 3 [1–4] median 3 [1–5] median 3 [1–5]	
Number of days into stay when penA focused history was taken (median (IQR))	2 [1–4]		
Day of inpatient stay penA risk assessment (median (IQR))	2 [1–5]		
Charlson Comorbidity Index (CCI) (median (IQR))	4 [2–6]		
Low risk penA history (n)	275 (55.3%)		
High risk penA history (n)	182 (36.6%)		
Unknown penA history (n)	40 (8.0%)		
LOS median (IQR)	9 (IQR 4.5–16)		
90-day readmission rate (from PADL date or if not PADL’s date of penA risk assessment)	142 (28.6%)		
90-day mortality (from PADL date or if not PADL’s date of penA risk assessment)	92 (18.5%)		

Of 497 risk assessed patients, 186 (37.4%) patients were eligible and consented for a DOCT or de-label on history alone; 58/186 (31.2%) via DOCT and 128/186 (68.8%) via DDL. Of these, 56/58 (96.6%) and 126/128 (98.4%) were successfully de-labelled via DOC and DDL, respectively. Of those tested by DOC the New Early Warning Score at the time of DOC was median 1 (IQR 0–2) [27]. Sixty patients declined PADL and a further 18 low risk patients were excluded for clinical reason (appendix B8).

### Impact of PADL on antibiotic use

Antibiotic exposure and antibiotic consumption (DDDs), when grouped by AwARe category, was not significantly different between patients who were de-labelled when compared to those not de-labelled (Table 5).

**Table 5:** Shows the impact of penicillin allergy de-labelling on antibiotic exposure (percentage patients exposed to each AwARe category) and consumption in defined daily doses

		Not delabelled	delabelled	*p*-value
n		300	181	
Age (mean (SD))		67.61 (16.66)	70.57 (15.15)	0.051
Gender (%)	F M	191 (63.7) 109 (36.3)	108 (59.7 73 (40.3)	0.436
Ethnicity (%)	Cornish Other/Unknown White	47 (15.7) 5 (1.7) 248 (91.2)	14 (7.7) 2 (1.1) 165 (82.7)	0.033
NEWS (mean (SD))		3.73 (3.01)	3.40 (2.60)	0.224
Charlson co-morbidity index (mean (SD))		4.26 (2.75)	4.51 (2.76)	0.344
LOS (mean (SD))		14.90 (17.44)	13.77 (16.33)	0.481
Days prior to risk assessment ± de-label		3.86 (6.17)	2.52 (3.57)	0.008
Days post risk assessment ± de-label		11.05 (14.88)	11.25 (15.85)	0.885
Organ (%)	Abdomen	70 (23.3)	44 (24.3)	0.154
	Other	39 (13.0)	33 (18.2)	
	Respiratory	89 (29.7)	52 (28.7)	
	Skin/soft	46 (15.3)	32 (17.7)	
	Urinary	56 (18.7)	20 (11.0)	
**Antibiotic exposure percentage of patients**		Not de-labelled	De-labelled	
Pre-risk assessment/de-label Access antibiotic exposure (number of patients (%))		221 (73.7)	126 (69.9)	0.392
Pre-risk assessment/de-label Reserve antibiotic exposure (number of patients (%))		23 (7.7)	13 (7.2)	0.987
Pre-risk assessment/de-label Watch antibiotic exposure (number of patients (%))		189 (63.0)	93 (51.4)	0.016
Post-risk assessment/de-label Access antibiotic exposure (number of patients (%))		230 (76.7)	140 (77.3)	0.952
Post-risk assessment/de-label Reserve antibiotic exposure (number of patients (%))		36 (12.0)	22 (12.2)	1.000
Post-risk assessment/de-label Watch antibiotic exposure (number of patients (%))		185 (61.7)	103 (56.9)	0.349
**Antibiotic exposure DDDs**		Not de-labelled	De-labelled	
Pre-risk assessment/de-label Access antibiotic consumption DDD (median [IQR])		1.73 [0.00, 4.00]	1.67 [0.00, 4.00]	0.644
Pre-risk assessment/de-label Reserve antibiotic consumption DDD (median [IQR])		0.00 [0.00, 0.00]	0.00 [0.00, 0.00]	0.876
Pre-risk assessment/de-label Watch antibiotic consumption DDD (median [IQR])		1.00 [0.00, 4.00]	0.50 [0.00, 3.00]	0.034
Post-risk assessment/de-label Access antibiotic consumption DDD (median [IQR])		4.50 [0.33, 11.00]	5.00 [0.33, 9.57]	0.986
Post-risk assessment/de-label Reserve antibiotic consumption DDD (median [IQR])		0.00 [0.00, 0.00]	0.00 [0.00, 0.00]	0.930
Post-risk assessment/de-label Watch antibiotic consumption DDD (median [IQR])		2.25 [0.00, 7.50]	2.00 [0.00, 8.00]	0.655

### Evidence of harm

Of all the patients who were eligible for DDL/DOC (*n* = 186) medical notes were reviewed between date of de-label until discharge (median 4 (IQR 2–11.5) days after DOC and median 6 (IQR 2–15) days after DDL). Of 58 DOCT patients 2 retained their penA record due to a potential reaction after DOCT (see Table 6). All reactions were determined as potential reactions by the clinical teams caring for the patients. One further DOCT patient self-reported diarrhoea but consented to penA record removal and for their GP records to be updated, and another patient reported a hot face and feeling warm which they attributed to their DOCT but after patient counselling consented to penA removal (PADL). Of 128 DDL patients 2 retained their penA status due to side effects (see Table 6), of the de-labelled patients, 75/128 (58.6%) were exposed to a penicillin during their inpatient stay. We were unable to locate e-notes, and therefore unable to follow-up, 1 DOCT and 7 DDL patients.

**Table 6:** Evidence of harm from penicillin allergy de-labelling (PADL) and penicillin exposure and non-PADL and non-penicillin antibiotic exposure

Patient group	patients (n)	missing e-notes (n)	Reported harm (n)	Retained penA status patients’ reaction details
DOCT	58	2	2/56 (3.6%)	• Urticarial rash day 5 piperacillin/tazobactam • Itchy skin < 24 hours post dose. Patient reports she usually has itchy skin when not taking antihistamines, which had been withheld.
DDL	127	7	2/120 (1.7%)	• Pruritic 2 hours post amoxicillin dose (nil reaction peri-op when had IV amoxicillin in theatre during this stay). Nil rash nil other symptoms. Changed to TMG and then experienced tingling lips. Has tolerated TMG on previous admission. Patient as a lot of healthcare-related anxiety • Self-reported rash (not seen by clinicians).
Non-PADL	311	12	9/299 (3.0%)	• AGEP ciprofloxacin, • oropharyngeal candidiasis doxycycline, • delayed rash metronidazole, • pain in legs metronidazole, • itchy rash meropenem, • maculopapular rash teicoplanin, • itchy exanthem teicoplanin and ciprofloxacin, • lips tingling wheezy levofloxacin, • hallucinations attributed levofloxacin
**Total**	**497**	**21**	**11/475 (2.3%**	

DOCT = direct oral challenge test, DDL = direct de-label

Of 311 penA patients with antibiotic prescriptions who had a penA assessment but were not de-labelled, notes for 12 were not found, the remainder were follow-up for a median of 5 days (IQR 1–13)) between the date if risk assessment and discharge. There was evidence of a potential antibiotic related adverse drug reaction in 9/299 (3.0%) patients (see Table 5). Of those not de-labelled,12/311 (3.9%) were exposed to a penicillin antibiotic during their stay (both intentional and unintentional exposure) but not de-labelled for a variety of reasons, primarily due to inability to obtain an accurate penA history or unable to consent to de-label.

### Communication with GP

Of the 182 patients successfully de-labelled as inpatients, 44 (24.2%) did not have penA on their GP Summary Care Record (SCR) prior to PADL and therefore communication of new penA status was not relevant for these patients. None the less, there was evidence of communication with the GP via the discharge letter to remove the penA record from GP records for 18/44 (40.9%) PADLs and no evidence of communication for 26/44 (59.1%) (appendix B9).

Of 138 de-labelled patients who had a penA documented in their GP records, there was evidence of communication from the hospital for GPs to update the penA status for 94/138 (68.1%) patients of which 42/94 (44.7%) were actioned by GP surgeries and 52/94 (55.3%) were not. Of the 42 patients without evidence of communication with the GP, 2 (4.8%) had their penA status updated and 39 (95.2%) did not.

### Process evaluation

The qualitative studies that inform the process evaluation are reported elsewhere [18, 19], but in summary, PADL was well supported by senior HCW groups [18]. The two AMS pharmacist PADL champions were described as accessible and supportive of ward teams to deliver PADL. Confidence in the safely of PADL and the benefits increased over the implementation period as staff became more familiar with PADL and saw firsthand the benefit of PADL for their patients, but prioritising PADL was challenging given competing clinical priorities.

Some participants who had agreed to PADL described having a good explanation of the risks and benefits of PADL and were grateful of the time HCWs had spent to explore their penA record and to remove it and reported trusting the HCW and the PADL process. For those that declined testing described being too overwhelmed to consider PADL. There was some evidence that provision of further information on the benefits of testing and reassurance of the low risk of reactions when exposed to penicillin, and more time to consider PADL might have persuaded some of these patients to agree to PADL. Better patient counselling of the benefits and risks of PADL may motivate more patients to agree to PADL. (See Table 7).

**Table 7:** Shows the key previously identified barriers to penicillin allergy de-labelling, a summary of key linked findings from the qualitative studies undertaken during the implementation of penicillin allergy de-labelling and a process evaluation of the implementation intervention

**Previously established Barriers to implementation.** [28, 29]	**Summary of key findings from the qualitative studies published elsewhere.** [18, 19]	**Process evaluation (interpretation of key findings from qualitative studies published elsewhere).** [18, 19]
Doctors, pharmacists MOPTs are not confident taking a penA focused history.	All the interviewed HCWs who had attended the training and also the MOPTs that had missed the formal training were confident taking a penA history. The middle grades that had not attended training felt they needed some training to increase their confidence with penA history taking.	The training successfully increased the knowledge and skills required to confidently take a penA history. The MOPTs that has missed the training were shown how to do it by the ward pharmacists working with them on the wards. That same sharing of knowledge was not seen amongst the doctors. Doctors who had missed the training were not then shown by ward doctors that had attended the training how to take a penA focused history.
PADL is not viewed as a priority.	PADL was viewed as important by all HCWs, but it was not viewed as a priority, but instead an aspiration when they had capacity.	The implementation intervention did raise awareness amongst HCWs that PADL was beneficial to patients but the specific benefits to patients were unknown except for one consultant who had learned about the benefits of PADL from an allergy colleague in another hospital. The education program failed to convince HCWs that PADL was a priority as PADL was often given a lower priority than other clinical tasks.
Not able to confidently differentiate low risk and high-risk penA histories.	The MOPTs and the pharmacists reported that their confidence risk stratifying patient built throughout the implementation period as they gained experience with PADL. The FY doctors said the training had given the skills and enough confidence to start PADL on the wards but that with experience their confidence would grow. Part 2 PADL training was designed to increase confidence with risk assessment and de-labelling but the uptake by pharmacists and doctors was low.	The training, education and ongoing AMS pharmacist champion PADL support all helped with instilling and building confidence to differentiate low and high risk penA records. Confidence will likely build further with HCW experience with PADL. The part 2 training, designed to build confidence with risk stratification, was poorly attended. Consideration with how to increase part 2 uptake may also more rapidly help with confidence building.
Pharmacist & doctor concerns about the safety of DOCT.	Although confidence increased over the study period there remained some safety concerns amongst the doctors and pharmacists, although they were balanced against the benefits of de-labelling.	The training did not do enough to dispel concerns about safety. Although all interviewees accepted that the befits outweighed the risks the perceived risk were making some HCWs hesitant to engage with PADL. With time and experiencing the low-risk nature of PADL themselves, confidence was expected to grow.
Senior support is required for more junior staff to de-label (DOCT).	All medical and surgical consultants engaged with the specialty meetings were supportive of the PADL intervention. Senior nurse and pharmacy support for PADL. Responsible clinicians were not declining DOCs because of safety concerns.	The specialty medical and surgical meetings, the senior nurse, the senior pharmacist and attendance at the MPC meeting all served their purpose in garnering senior clinical staff support for PADL.
A lack of PADL champions	Two PADL champions, both antimicrobial pharmacists, engaged and actively leading the delivery of PADL within the organisation. The PADL champions were said to be accessible, supportive and facilitated the ward teams with PADL.	The PADL champions were vital in both delivering PADL and modelling PADL for other healthcare workers, encouraging their involvement, but also with ongoing PADL support for ward doctors and ward pharmacists, supporting their decision making and facilitating PADL through prescribing of DOC, when required.
PADL is not currently supported by the organisation.	PADL guidelines agreed by Medicines Practice Committee and published on the hospital intranet.	The PADL guidelines were published prior to the launch of the implementation intervention and made available via the intranet. Engagement with the guidelines was poor, overall. It is unclear how much of an impact poor accessibility had on the delivery of the intervention.
There needs to be senior nurse support to deliver target behaviour	Senior nurse support for PADL was pledged. We were unable to recruit nurses to the qualitative interviews and therefore unable to determine whether the message had been cascaded to nurses. There were no posters visible in ward areas.	We were unable to formally evaluate this domain. AMS champion pharmacists did not experience any reluctance from nurses to administer the DOCT nor to undertake the required observations. Our previous qualitative study exploring HCW views on a theoretic inpatient PADL pathway, which included views from nurses, nurses expressed that that their role in PADL would be deliverable [28]
There are competing demands on nurse time.		
Patient counselling is important.	Patients de-labelled by DOC reported they had received adequate explanation about the risks and benefits of PADL whereas the patients de-labelled by DDL were vaguer about the explanations they had received, suggesting those de-labelled by DOC were given a more thorough explanation. Of those that refused PADL, some said that an outpatient testing appointment would be more agreeable to them and some said they had not had an adequate explanation of the risks and benefits and of the process and would agree to PADL if offered again, but for those patients they still expressed some anxiety about being de-labelled. We were unable to de-label 60 patients because they declined either DOC or DDL.	Patient anxiety about the safety of testing remains a barrier for some patients. The counselling patients received was largely reported as adequate and that they were provided with adequate information to enable them to make the decision to decide to go ahead with PADL. For others, the information received was not enough to help them make the decision to go ahead with PADL. For others their anxiety and their perceived acuity of illness was preventing them from agreeing to PADL and likely these patients will not be persuadable in the inpatient setting during their acute episode of illness.
Patients unaware of the negative consequences of penA records & have concerns about safety of testing.		

[18, 19, 28, 29]. MOPTs = medicines optimisation pharmacy technicians, penA = penicillin allergy, PADL = penicillin allergy de-labelling, DOCT = direct oral challenge tes

### Costs of PADL

#### Part 1 PADL training

Training costs were calculated accounting for both staff time to complete training and to deliver training (i.e. both trainers and trainees) which were £2,127 to deliver part 1 training to 15 MOPTs, 116 FY1&2 doctors and 37 pharmacists. (For full breakdown of PADL costs see appendix C.)

#### Part 2 PADL training

To deliver the 40-minute part 2 PADL training session to 10 pharmacists and 4 doctors cost £615.69.

#### Specialty engagement meetings

PADL champion time (15 minutes per meeting) costed to attend 12 specialty meetings was £141.09. Meeting attendees time has not been costed because meeting attendees were not recorded.

#### Staff costs to document penA histories (add PADL notes to EPMA)

Cost of the 1115 PADL notes over the study period using the salary costs of those taking the penA history for an average 5 minutes per penA history was £1,573.58

#### Staff costs to de-label by either DOC or DDL

To de-label 58 patients by DOC cost £1307 in staff resource (£22.53 per patient), 128 by DDL cost £328.87 (£2.57) per patient.

### Total costs to de-label

Staff resource to de-label the 186 patients, which included training and engagement meetings, taking the penicillin allergy history for 1115 patients and to de-label eligible patients (which includes counselling, prescribing the test dose, delivering the enhanced observations and communication with the GP) was £7131.01, or £38.34 per patient de-labelled. If the set-up costs of training and engaging meetings are excluded the staff resource cost to de-label one patient was £17.26.

## Discussion

### Summary of findings

There was a marked increase in PADL compared to baseline levels suggesting the implementation was successful. There was a successful expansion of the workforce competent in PADL as over half the penA histories were taken by the ward clinical teams, predominantly pharmacy staff as part of the medicine’s reconciliation process. Doctors considered pharmacists as well placed to deliver and to lead PADL. Most patients were de-labelled by the AMS pharmacists, but patients were also de-labelled by ward doctors, ward pharmacists and one by a MOPT. PADL was delivered across a broad range of clinical specialties in the hospital with marked variation across specialties.

There was evidence of written communication on the patient’s new de-labelled status from the hospital to the GP for most patients, but we identified many patients without communication. Of those with GP communication just under half had had their penA records updated on the GP system whereas it was even worse (5%) where there was no evidence of written communication with the GP.

More than a third of risk assessed patients were de-labelled, predominantly on history alone. Nearly a quarter of patients declined de-label with the majority not providing reasons for declining PADL. PADL was not associated with any changes in Access or non-Access antibiotic prescribing. The rates of antibiotic associated adverse drug events were low, and comparable across the two de-labelled patient groups and the non-de-labelled group. However, there were statistically significant differences between the delabelled and non delabelled populations, meaning that comparisons between the two may not have been an ideal method to assess prescribing changes.

### Strengths and limitations

We followed an integrated approach to develop a behavioural change implementation intervention that combined theory-based, evidence based, and person-based approaches designed to support non-allergist healthcare workers to remove incorrect penA records from medical and surgical adult in patients. This thorough and robust development process gave the implementation intervention a good chance of successfully being implemented.

The study was single centre which may limit its generalisability to other hospitals. There has been considerable previous work on PADL in the study hospital that may affect transferability. Our surveillance method may have missed some patients, for example, patients de-labelled by ward teams prior to documenting their penA status on EPMA, patients de-labelled with a short inpatient stay or those admitted and discharged out of hours or over a weekend.

Patients were followed up while inpatients for this episode, therefore follow up was short and there are likely to be additional benefits of delabelling over time that we have not collected. We did not assess the clinical outcome of patients. Our control group for assessing impact on prescribing differed significantly from the de-labelled patients.

The costs for the staff time are true if PADL was incorporated as part of standard of care, i.e. when patients were seen as part of routine rounds. However, delivering PADL as a dedicated ward round is likely more expensive than the costs presented here due to the time it takes to locate paper hospital notes, accessing patients, and to locate senior consultants to obtain permission to prescribe a DOCT. We were unable to cost clinician attendance at their regular specialty meetings used by the PADL champion to raise awareness of the proposed PADL intervention. However, the attendance was low and the discussion short (10 minutes) and therefore these costs were low.

### Comparisons with other studies

Blumenthal et al. developed an allergist-led beta-lactam de-labelling implementation intervention in a group of US hospitals in 2016 [30, 31]. Similar to our study, Blumenthal engaged key stakeholders, developed de-label guidelines, a patient pathway and provided education to providers in the key antimicrobial prescribing specialties [30]. Blumenthal et al. evaluated the impact of the education delivered to providers on PADL knowledge using a pre and post education survey and found that post education providers reported that they were interested in using the PADL guidelines to de-label patients, felt prepared to risk assess penA and felt more prepared to prescribe beta-lactams in patients with penA [32]. Likewise, doctors and pharmacists reported the education we provided gave them the knowledge and the confidence required to engage with PADL on their wards [18]. Blumenthal et al. were able to integrate the guidelines and PADL prompts within the electronic health records as a ‘best practice alert’ (BPA), which we were unable to do with our electronic prescribing system [30]. We were able to make the PADL tools easily accessible to pharmacy teams through a shared electronic folder accessed daily by pharmacy teams but we were unable to provide doctors with such easy access, which might have contributed to their lower engagement with PADL when compared to pharmacy staff. In our process evaluation, doctors reported that incorporating the PADL tools into the EHR with prompts to de-label patients would likely increase doctor engagement with PADL [18]. Blumenthal et al. encouraged general medical teams to perform DOCT doses on general inpatient units. A monthly reporting dashboard including key data for ongoing assessment and monitoring was created and included the following process measures; web site usage, web site traffic, and BPA alert firing [30]. Over 21 months there were 1,046 test doses, predominantly cephalosporins, prescribed mainly by house staff and in internal medicine, across 5 hospitals, with allergist support for only 9% of test doses. After the publication of their PADL guidelines and education sessions on how to use the guidelines and deliver PADL the rates of DOC test doses increased 7-fold in ward areas [33]. Patients were in the hospital a median of 2 days (IQR; 1–4 days) prior to their test, similar to our findings. The rate of ADRs was higher than our cohort at 7.5% with several requiring treatment including adrenaline and corticosteroids.

More recently Brayson et al. published their PADL pilot delivered by 12 UK clinical ward non-medical prescribing pharmacists, led by the AMS team, which included antimicrobial pharmacists and an infectious disease and microbiology consultants [34]. The AMS team developed local PADL guidelines utilising the SAPG testing protocol and PEN-FAST [35]. All ward staff were informed about the PADL processes before PADL was piloted. The manuscript does not describe further implementation strategy such as education provided and whether prompts were required or the role of PADL champions [34]. Over a 10-month pilot, 304 penA patients were risk assessed, 132 (43%) were de-labelled; 73 by DOC and 59 by DDL with 1 (0.76%) patient having a serious ADR (interstitial nephritis) [34]. GP records were amended with the new allergy status for just 52% of de-labelled patients which increased to 64% following further work to improve that step [34]. Our project also suffered from poor rates of transfer of de-labelling status to primary care records, a major flaw that threatens to undermine the impact of de-labelling for many patients. There are electronic health record system approaches that can improve this as 96% of GP records were updated in the ALABAMA trial and this needs to be incorporated into future interactions of the intervention [8].

Previously, we reported the findings of a systematic review of non-allergist delivered PADL and found across the studies the de-label rate for DOC was 27% and DDL was 14% (combined 41%) and the rate of harm was reported as nil for DDL and1% for DOC [6]. Overall combining DDL and DOC our rate of de-label was comparable to the SR findings with 37% of patients risk assessed and de-labelled; DDL 25.8% and DOC 11.3% but the proportion DDL was higher in our study [6]. We found a higher rate of harm associated with DOC in our study when compared to our SR findings but comparable to the rate of harm found in a larger SR of 9225 patients of 3.5%, and like others the reactions reported in our study were mild or subjective [6, 36]. Contrary to the nil reported associated side effects reported post DDL in the systematic review by Powell et al. we found a low rate of associated harm which was comparable to the group not de-labelled [6]. The rate of PADL reported by Brayson et al. was similar to our findings but the majority of our patients were de-labelled by DDL and not DOCT as per Brayson et al [34]. The reported harm by Brayson was lower than our study with only one patient (0.76%) having an ADR, but unusually for de-label studies it was a serious ADR (possible interstitial nephritis) [34].

The wider literature reports a range of HCWs involved in PADL which, like our study, includes doctors and pharmacists. MOPTs were key in our intervention, documenting penA histories as part of the medicine’s reconciliation process, and the involvement of this staff group has not been reported by others. We found that MOPTs, with support and further training, were amenable to expand their role to include DDL [18]. The potential role of MOPTs, as well as pharmacists, in PADL has been recognised by others [37]. The role of a PADL champion is not well reported in the literature. Most non-allergist delivered studies are delivered by either an individual or a small team of infection or AMS experts, with few reporting similar models to ours which was championed by AMS pharmacists who both delivered PADL and encouraged and supported the wider healthcare team to do the same [6]. The champion role was key in our implementation intervention and model of PADL delivery and identified as key in other implementation interventions [38].

The cost of de-labelling 355 Australian inpatients (161 DDL and 194 DOCT) was reported to be $6825 (£3,269.86) or $35.18 Australian dollars (£18.38) per DOC patient and no cost implications reported for DDL patients [39]. Our costs to de-label are comparable to the Australian inpatient costs at £22.53 per DOCT patient, £2.57 per DDL patient. A UK inpatient PADL study delivered by either a pharmacist or a nurse estimated the cost of DOCT to be much higher at £577 per haematology-oncology inpatient and £2329 per AMU/infectious diseases patient [40]. Their larger costs were explained by healthcare worker time required to screen the large numbers of patients to de-label the small numbers. Only 3.3% of screened inpatients were de-labelled compared to the Australian study de-label rate of 29.8% and our de-label rate of 37.4%.

We found PADL was associated with an increase in ‘Access’ antibiotic prescribing but not a reduction in non-Access (Watch and Reserve) prescribing. This did not reach statistical significance when compared to the convenience control group of non-de-labelled patients. Anton-Vazquez et al. found penA patients in a UK hospital more frequently received antimicrobials from the non-Access groups and those de-labelled more often received antimicrobials from the ‘Access list’ (63% vs 8%; *p* < 0.001) [41]. The reasons for our study not showing an impact of PADL on Access prescribing is likely due to our increased Access antibiotic prescribing prior to the intervention, the small sample size and short follow-up period. Previously, in a modelling study using local inpatient prescribing data, we estimated that de-labelling 50% of penA patients would reduce non-Access antibiotic use by 5.8% and total antibiotic use by 0.86% [5]. Since then, the hospital antimicrobial guidelines have been revised and updated to reduce prescribing of antimicrobials from the non-Access groups. Local use of Access antibiotics as a proportion of non-Access antibiotics has increased from 57% in 2019 to 69% in 2024 indicating that our background use of Access antibiotics is now higher compared to the modelling data period and as such patients with penA records are already receiving a high proportion of Access antibiotics first line (unpublished data). In our current analysis we found that exposure to the Access group was high with over 70% exposure to Access antibiotics in both the patients de-labelled and not de-labelled prior to risk assessment. Understanding which antibiotics are driving those differences and for which infections might explain the low impact of PADL on our antibiotic use when measured using AWaRe categories. We included all patients with a penA record and prescribed antibiotics, regardless of whether the de-label would then prompt an immediate change in antibiotic. Mir et al. included the same cohort for PADL and they did find an impact of PADL on antibiotic prescribing but the measure was changes in beta-lactam use rather than AWaRe categories [42].

### Conclusions and implications

We have implemented a PADL intervention and made progress expanding the non-specialist workforce able to assess patients with low risk PenA histories. Pharmacy champions and MOPTs were found to be key components of the team.

There is opportunity to further optimise PADL through encouraging ward pharmacy teams to standardise penA history taking as part of medicine reconciliation and ensuring more patients are risk assessed. Delivering PADL as part of routine care instead of dedicated ward rounds is likely more cost effective. We have identified a need to further improve the communication of new allergy status with GP records, essential in the prevention of re-labelling when re-admitted as well as optimising GP antibiotic prescribing and better communication with patients to convince them of the safety of PADL.

The impact of PADL on non-Access and total antibiotic use is trending in a positive direction but further data are required to see if PADL is having a statistically significant impact on AMS using this high-level AMS metric.

## Electronic supplementary material

Below is the link to the electronic supplementary material.

Supplementary Material 1

Supplementary Material 2

Supplementary Material 3

Supplementary Material 4

Supplementary Material 5

## Data Availability

Data available on request.

## References

[1] Krah NM, Jones TW, Lake J, Hersh AL. The impact of antibiotic allergy labels on antibiotic exposure, clinical outcomes, and healthcare costs: a systematic review. Infect Control Hosp Epidemiol. 2021;42(5):530–48. 10.1017/ice.2020.1229. 33059777 10.1017/ice.2020.1229

[2] Powell N, Honeyford K, Sandoe J. Impact of penicillin allergy records on antibiotic costs and length of hospital stay: a single-centre observational retrospective cohort. J Hosp Infect. 2020;106(1):35–42. 10.1016/j.jhin.2020.05.042. 32502582 10.1016/j.jhin.2020.05.042

[3] DesBiens M, Scalia P, Ravikumar S, Glick A, Newton H, Erinne O, et al. A closer look at penicillin allergy history: systematic review and meta-analysis of tolerance to drug challenge. Am J Med. 2020;133(4):452. 10.1016/j.amjmed.2019.09.017. 31647915 10.1016/j.amjmed.2019.09.017

[4] World Health Organisation. WHO releases the 2019 AWaRe classification antibiotics 2019 [Available from: https://www.who.int/medicines/news/2019/WHO_releases2019AWaRe_classification_antibiotics/en/.

[5] Powell N, West R, Sandoe JAT. The impact of penicillin allergy de-labelling on the WHO AWaRe antibiotic categories: a retrospective cohort study. J Hosp Infect. 2021;115:10–16. 10.1016/j.jhin.2021.04.011. 33895164 10.1016/j.jhin.2021.04.011

[6] Powell N, Stephens J, Kohl D, Owens R, Ahmed S, Musicha C, et al. The effectiveness of interventions that support penicillin allergy assessment and delabeling of adult and pediatric patients by nonallergy specialists: a systematic review and meta-analysis. Int J Infect Dis. 2023;129:152–61. 10.1016/j.ijid.2022.11.026. 36450321 10.1016/j.ijid.2022.11.026 PMC10017351

[7] Sacco KA, Bates A, Brigham TJ, Saadi-Imam J, Burton MC. Clinical outcomes following inpatient penicillin allergy testing: a systematic review and meta-analysis. Allergy. 2017;72(9):1288–96. 10.1111/all.13168. 28370003 10.1111/all.13168

[8] Sandoe Jat AS, Armitage K, et al. Penicillin allergy assessment pathway to safely increase penicillin use. Lancet Primary Care. 2025, In press.

[9] Sandoe JAT, Ahmed S, Armitage K, Bates C, Bestwick R, Boards J, et al. Penicillin allergy assessment pathway versus usual clinical care for primary care patients with a penicillin allergy record in the Uk (ALABAMA): an open-label, multicentre, randomised controlled trial. LancetThe Lancet Primary Care.

[10] Skivington K, Matthews L, Simpson SA, Craig P, Baird J, Blazeby JM, et al. A new framework for developing and evaluating complex interventions: update of medical Research council guidance. BMJ. 2021;374:n 2061. 10.1136/bmj.n2061. 10.1136/bmj.n2061 PMC8482308 34593508

[11] O”Cathain A, Croot L, Duncan E, Rousseau N, Sworn K, KM, et al. Guidance on how to develop complex interventions to improve health and healthcare. BMJ Open. 2019;9(8):e029954. 10.1136/bmjopen-2019-029954. 10.1136/bmjopen-2019-029954 PMC6701588 31420394

[12] Yardley L, Morrison L, Bradbury K, Muller I. The person-based approach to intervention Development: application to digital Health-related behavior change interventions. J Med Internet Res. 2015;17(1):e30. 10.2196/jmir.4055. 10.2196/jmir.4055 PMC4327440 25639757

[13] Eldh AC, Almost J, DeCorby-Watson K, Gifford W, Harvey G, Hasson H, et al. Clinical interventions, implementation interventions, and the potential greyness in between -a discussion paper. BMC Health Serv Res. 2017;17(1):16. 10.1186/s12913-016-1958-5. 28061856 10.1186/s12913-016-1958-5 PMC5219812

[14] Neil Powell MU, Bridie KENT, Jonathan SANDOE, Tonkin-Crine S. Description of the combined evidence based, theory-based and person-based approaches used to develop a behavioural intervention package to support non-allergist healthcare workers to remove incorrect penicillin allergy records from medical and surgical adult inpatients in a Uk hospital. In: BMJ open. *In Press*; 2025. 10.1136/bmjopen-2024-096452 PMC12315033 40738640

[15] Damschroder LJ, Reardon CM, Opra Widerquist MA, Lowery J. Conceptualizing outcomes for use with the Consolidated framework for implementation Research (CFIR): the CFIR outcomes addendum. Implementation Sci. 2022;17(1):7. 10.1186/s13012-021-01181-5. 10.1186/s13012-021-01181-5 PMC8783408 35065675

[16] Curran GM, Bauer M, Mittman B, Pyne JM, Stetler C. Effectiveness-implementation hybrid designs: combining elements of clinical effectiveness and implementation research to enhance public health impact. Med Care. 2012;50(3):217–26. 10.1097/MLR.0b013e3182408812. 22310560 10.1097/MLR.0b013e3182408812 PMC3731143

[17] Forman J, Heisler M, Damschroder LJ, Kaselitz E, Kerr EA. Development and application of the RE-AIM QuEST mixed methods framework for program evaluation. Prev Med Rep. 2017;6:322–28. 10.1016/j.pmedr.2017.04.002. 28451518 10.1016/j.pmedr.2017.04.002 PMC5402634

[18] Neil Powell N, Upton M, Kent B, Sandoe JAT, Tonkin-Crine S. Assessing a penicillin allergy de-labelling implementation intervention in a Uk hospital: a process evaluation reporting healthcare workers’ experiences. JAC Antimicrob Resist. 2025, In press.7(5). 10.1093/jacamr/dlaf174. 10.1093/jacamr/dlaf174 PMC12509609 41079144

[19] Powell N, Upton M, Kent B, Sandoe JAT, Tonkin-Crine S. Penicillin allergy de-labelling implementation intervention in a Uk hospital: a process evaluation, the patient experience. JAC Antimicrob Resist. 2025;7(4):dlaf144. 10.1093/jacamr/dlaf144. 10.1093/jacamr/dlaf144 PMC12344413 40809340

[20] Pinnock H, Barwick M, Carpenter CR, Eldridge S, Grandes G, Griffiths CJ, et al. Standards for reporting implementation studies (StaRI) Statement. BMJ. 2017;356:i 6795. 10.1136/bmj.i6795. 10.1136/bmj.i6795 PMC5421438 28264797

[21] Powell N, Elkhalifa S, Sandoe J. Penicillin allergy de-labelling by non-allergists: a comparison of testing protocols. JAC Antimicrob Resist. 2023;5(6):dlad134. 10.1093/jacamr/dlad134. 10.1093/jacamr/dlad134 PMC10729857 38115860

[22] Powell N, Elkhalifa S, Hearsey D, Wilcock M, Sandoe J. The appropriateness of penicillin allergy de-labelling by non-allergist clinical ward teams. Clin Med (Northfield Il). 2024;24(4):100225. 10.1016/j.clinme.2024.100225. 10.1016/j.clinme.2024.100225 PMC11304015 38944245

[23] Powell N, Upton M, Kent B, Tonkin-Crine S, Sandoe J. Is penicillin allergy de-labelling about to find its place in Uk antimicrobial stewardship strategy? Clin Med (Northfield Il). 2023;23(1):76–77. 10.7861/clinmed.2022-0518. 10.7861/clinmed.2022-0518 PMC11046535 36650063

[24] Hearsey D, Elkhalifa S, Sandoe J, Wilcock M, Owens R, Gay B, et al. Removal of incorrect penicillin allergy labels in a Uk hospital. Clin Microbiol Infect. 2023;29(10):.e1338.1–.4. 10.1016/j.cmi.2023.06.024. 10.1016/j.cmi.2023.06.024 37354996

[25] NIfHac E. Medicines optimisation 2016. Updated 24th. 2016. Available from: March. https://www.nice.org.uk/guidance/qs120/resources/medicines-optimisation-pdf-75545351857861.

[26] Braun V, Clarke V. Conceptual and design thinking for thematic analysis. Qualitative Phychol. 2022;9(1):3–26. 10.1037/qup0000196.

[27] Rco P. National early warning score (NEWS) 2 2017 [Available from: https://www.rcp.ac.uk/improving-care/resources/national-early-warning-score-news-2/.

[28] Powell N, Upton M, Kent B, Sandoe J, Tonkin-Crine S. Non-allergist healthcare workers views on delivering a penicillin allergy de-labelling inpatient pathway: identifying the barriers and enablers. JAC Antimicrob Resist. 2023;6(1). 10.1093/jacamr/dlae014. 10.1093/jacamr/dlae014 PMC10848892 38328264

[29] Powell N, Upton M, Kent B, Sandoe J, Tonkin-Crine S. Experiences of an inpatient penicillin allergy de-labelling pathway: capturing the patient voice. JAC Antimicrob Resist. 2023;6(1):dlae020. 10.1093/jacamr/dlae020. 10.1093/jacamr/dlae020 PMC10854212 38343626

[30] Blumenthal KG, Wickner PG, Hurwitz S, Pricco N, Nee AE, Laskowski K, et al. Tackling inpatient penicillin allergies: assessing tools for antimicrobial stewardship. J Allergy Clin Immunol Pract. 2017;140(1):154–61.e6. 10.1016/j.jaci.2017.02.005. 10.1016/j.jaci.2017.02.005 PMC5496780 28254470

[31] Blumenthal KG, Wolfson AR, Hsu JT, Shenoy ES, Li Y, Schwartz JM, et al. Outcomes of beta-lactam antibiotic test dose procedures for patients with reported beta-lactam allergy performed in a large US healthcare system. J Allergy Clin Immunol Pract. 2019;143(2):AB25. 10.1016/j.jaci.2018.12.078.

[32] Blumenthal KG, Shenoy ES, Hurwitz S, Varughese CA, Hooper DC, Banerji A. Effect of a drug allergy educational program and antibiotic prescribing guideline on inpatient clinical providers’ antibiotic prescribing knowledge. J Allergy Clin Immunol: Pract. 2014;2(4):407–13. 10.1016/j.jaip.2014.02.003. 25017528 10.1016/j.jaip.2014.02.003 PMC4309264

[33] Blumenthal KGMD, Shenoy S, Varughese CPD, Hurwitz SP, Hooper DMD, Banerji AMD. Impact of a clinical guideline for prescribing antibiotics to inpatients with reported penicillin or cephalosporin allergies. J Allergy Clin Immunol Pract. 2015;135(2):AB232–AB. 10.1016/j.jaci.2014.12.1694.

[34] Brayson J, Barrett S, Baqir W, Campbell D, Oswald T, Ellis S, et al. CATALYST: challenging antibiotic allergy status. The J Antimicrob Chemother. 2023;78(5):1241–44. 10.1093/jac/dkad081. 36975000 10.1093/jac/dkad081

[35] Trubiano J, Vogrin S, Chua K, Bourke J, Yun J, Douglas A, et al. PEN-FAST: a validated penicillin allergy clinical decision rule – implications for prescribing. Int J Infect Dis. 2020;101(Supplement 1):89. 10.1016/j.ijid.2020.09.259.

[36] Blumenthal KG, Smith LR, Mann JTS, Salciccioli I, Accarino JJO, Shah RJ, et al. Reaction risk to direct penicillin challenges: a systematic review and meta-analysis. JAMA Intern Med. 2024;184(11):1374–83. 10.1001/jamainternmed.2024.4606. 39283610 10.1001/jamainternmed.2024.4606 PMC11406457

[37] Gray MP, Dhavalikar N, Boyce RD, Kane-Gill SL. Qualitative analysis of healthcare provider perspectives to evaluating beta-lactam allergies. J Hosp Infect. 2023;141:198–208. 10.1016/j.jhin.2023.07.024. 37574018 10.1016/j.jhin.2023.07.024

[38] Urquhart R, Kendell C, Geldenhuys L, Ross A, Rajaraman M, Folkes A, et al. The role of scientific evidence in decisions to adopt complex innovations in cancer care settings: a multiple case study in Nova Scotia, Canada. Implementation Sci. 2019;14(1):14. 10.1186/s13012-019-0859-5. 10.1186/s13012-019-0859-5 PMC6371509 30755221

[39] Chua KYL, Vogrin S, Bury S, Douglas A, Holmes NE, Tan N, et al. The penicillin allergy delabeling program: a multicenter whole-of-hospital Health services intervention and comparative effectiveness study. Clin Infect Dis. 2021;73(3):487–96. 10.1093/cid/ciaa653. 32756983 10.1093/cid/ciaa653 PMC8326579

[40] Bestwick R, Bhogal R, Kildonaviciute K, Ng BY, Jackson B, Moriarty C, et al. An economic evaluation of direct oral penicillin challenge for De-labelling low risk patients with a penicillin allergy label. Clin Exp Allergy. 2025;55(5):378–90. 10.1111/cea.14633. 39909457 10.1111/cea.14633 PMC12088836

[41] Anton-Vazquez V, Ferretti F, Kaya D, Mishra S, Kerneis S, Eden C, et al. Impact of penicillin allergy records on antimicrobial prescribing in hospitalised patients. Clin Med (Northfield Il). 2024;24(2):100024. 10.1016/j.clinme.2024.100024. 10.1016/j.clinme.2024.100024 PMC11091395 38382835

[42] Mir A, Lanoue D, Zanichelli V, van Walraven C, Olynych T, Nott C, et al. Introduction of a penicillin allergy de-labelling program with direct oral challenge and its effects on utilization of beta-lactam antimicrobials: a multicenter retrospective parallel cohort study. Allergy Asthma Clin Immunol. 2024;20(1):20. 10.1186/s13223-024-00877-9. 38444037 10.1186/s13223-024-00877-9 PMC10913637

