# Supplementary material for: Outcomes of a penicillin allergy de-labelling implementation intervention in a UK hospital: a prospective, single centre, type 2 hybrid effectiveness-implementation study evaluated using mixed methods
Source: Implement Sci Commun. 2026 Jun 11;7:157. doi: 10.1186/s43058-026-01008-8 (PMC13488184; doi:10.1186/s43058-026-01008-8)
Supplement: Supplementary file 1 [file 43058_2026_1008_MOESM2_ESM.docx]

|  | **Key elements within the phase** | **Evidence for meeting phase requirements** | **Phase requirements not met** | **comments / plans** |
| --- | --- | --- | --- | --- |
| **Phase 1: identify the practice area**  Penicillin allergy de-labelling delivered by non-allergy healthcare workers | It is important that the practice area identified is relevant and of interest to the project team (who are conducting the evidence implementation). | The project team outside the organisation are interested in PADL (PhD supervisory team).  PADL is relevant and of interest to the local implementation team, Neil Powell and Daniel Hearsey, both antibiotic pharmacists. |  |  |
|  | It is important that the practice area identified is relevant and of interest to the organization (in which the project is being conducted). | PADL approved by the hospitals Medicines Practice Committee co-chaired by the deputy Chief Medical Officer & Chief Pharmacist. Antibiotic stewardship committee approval. | nil |  |
|  | It is important that the practice area identified is relevant and of interest to the clinical team (whom the practice improvement initiative/evidence implementation project will impact) | Medical teams - letters / emails of support for PADL received by all medical specialties. Three-month PADL pilot study, delivered by pharmacists and junior doctors in medical specialties and in surgical specialties was well received by clinicians in all areas where patients were de-labelled, evidenced by consultants agreeing to testing before we went ahead with PADL. Only one respiratory physician denied consent to test their patient. Qualitative in-depth interviews with doctors (Consultants to ED/acute medicine/eldercare, middle grade medical doctors and Foundation Year doctors) showed PADL to be relevant to their practice and of interest. Ward pharmacists, ward pharmacy technicians, ward nurses and senior nurses were all supportive and felt PADL of interest to them. A focus group study and a survey study of doctors, nurses and pharmacists working at the RCHT showed that the majority thought penA was a problem that needed a solution. PADL approved at medical and surgical specialty governance meetings during the re-launch in June 2024. | nil |  |
|  | It is important that the practice area identified is relevant and of interest to patients | PPIE with inpatients, a face-to-face PPIE focus group and online small focus group with patients with penA labels reveal that patients would like to be tested, and where able, their penA label removed. The 3-month pilot study in medical and surgical specialties enabled the identification of patients with penA labels that were de-labelled and those that weren't and their views on PADL were explored in one-to-one interviews. Although there was reluctance / refusal to testing by some inpatients, a large proportion of patients agreed to testing. As part of the ALABAMA study >100 patient from one Truro GP practice came forward for penA testing, again demonstrating a desire from patients to be tested. | nil |  |
| **Phase 2: engage change agents** - "individual or group that undertakes the task of initiating and managing" Change agents may be leaders, facilitators, or clinical champions; their involvement is pivotal to successful change. Different kinds of change agents exist and knowing these can assist in identifying whom to inform, whom to engage, and whom to invite as active members of the implementation project group. | **System leadership**: This person is an influencer and leads across departments, organizations, or sectors. System leaders can overcome barriers and facilitate progress towards change. | Neil Powell (Consultant Antimicrobial Pharmacist Associate Director AMS, Director AMS ICS). | nil |  |
|  | **Technical expertise**: This person has exceptional knowledge of the subject matter, which requires a degree of enthusiasm and expertise. If the subject is particularly broad, such as vital signs, technical experts with different backgrounds are warranted | Neil Powell has requisite enthusiasm and PADL expertise. | nil |  |
|  | **Day-to-day leadership**: This responsibility should rest with someone who has time and professional interest or enthusiasm to devote to the project. This person should be able to recruit others and encourage relevant activities. | Neil Powell has requisite enthusiasm and PADL expertise, time and professional interest to devote to the project. | nil |  |
|  | it is crucial to set up an implementation project group and appoint a project lead whose role is to coordinate the group, clarify the role and responsibilities of those involved in the project, provide access to EBHC resources, and oversee all aspects of the implementation project. | The local implementation Neil Powell and Daniel Hearsey. | nil |  |
| **Phase 3: assess context and readiness to change** Undertaking a contextual analysis and assessment of readiness to change is a key component of the initial phase of any implementation project. Context analysis is a diagnostic process, the purpose of which is to understand issues within the local context that are important to practice change, and to identify factors likely to influence the proposed change | 1. Structure: To what extent does decision-making occur in a decentralized manner, and are there enough staff to support the change process? | Decision making is de-centralised. Whether there are enough staff to support change process is a good question! I believe yes, to an extent. | nil |  |
|  | 2. Workplace culture: To what extent is the proposed change consistent with the values and beliefs of the practice environment, and to what degree does the culture support change and value evidence? | I believe the culture supports change, and I believe the change is consistent with values of the healthcare workers delivering the intervention. | nil |  |
|  | 3. Communication: Are there adequate communication systems to support information exchange related to the change and implementation processes? | There are three options to communicate the test result with the GP, which are clear in the Trust guideline and also included in education and training on PADL: 1) (preferred method) use the letter template in the hospital PADL guideline and email to the GP surgery (2) Use EPMA note ‘note to appear in discharge letter, (3) include PADL details in the discharge letter. | Nil |  |
|  | 4. Leadership: To what extent do leaders within the practice environment support (visibly and behind the scenes) implementation? | Deputy Medical Director, Chief Pharmacist, Clinical Pharmacy Manager, Medical, surgical and critical care consultants are all supportive. | nil |  |
|  | 5. Resource availability: Are necessary human, physical, and financial resources available to support implementation? | The intervention is deliverable with the current human, physical and financial resource. | nil |  |
|  | 6. Knowledge, skills, and attitudes: Do staff members have the necessary knowledge and skills? Which potential groups are open to change and new ideas, and to what extent are they motivated to implement the change? | Doctors, nurses, medicines optimisation pharmacy technicians and pharmacists are motivated to implement change where the behaviour aligns with their role. There is a requirement for knowledge sharing and upskilling generally across the workforce, more so with the more junior members of staff within ach staff group. Awareness raising was well delivered required across all staff groups and to all staff levels with the exception of middle grade doctors / registrars. | Education and awareness raising to registrars required. |  |
|  | 7. Commitment to quality management: Do quality processes and systems exist to measure the implementation results? | Data will be collected on patient outcomes and appropriateness of penicillin allergy risk assessment to ensure that risk assessment is done in line with hospital guidelines and the risk stratification tool. Learning will be fed back promptly to teams. | nil |  |
|  | 8. Interdisciplinary relationships: Are there positive relationships and trust between disciplines that will be involved or affected by the change? | There are positive relationships between the medical teams, surgical teams, nursing teams and AMS (pharmacist and medical microbiology) and ward pharmacy teams. | nil |  |
|  | Cultural changes in the workplace may also be needed to facilitate the implementation of evidence. | The culture aligns with the intervention | nil |  |
| **Phase 4: review practice against evidence based.** Implementation is grounded in clinical audit and feedback using evidence-based audit criteria audit criteria (aka Quality Improvement). | Audit cycle includes baseline audit of practice, feedback of results to healthcare staff, reflection on barriers and potential strategies to address, then follow-up data collection to enable comparisons with the baseline data. | Ongoing audits to capture total number of penA patients discharged from hospital who have been allergy assessed and numbers de-labelled. | nil |  |
|  | Evidence based audit criteria to be established. | Total number of penA patients de-labelled by the AMS team and separately by clinical teams | nil |  |
|  | Audit plan drawn up so that it is continuous and used to embed PADL and used to reflect on barriers to implementation. | Identify clinical teams that are not delivering PADL and offer further support in these areas | nil |  |
| **Phase 5: implement changes to practice using Getting Research into Practice** Baseline audit to identify barriers and enablers to implementation change and to plan implementation change. Need to identify modifiable and non-modifiable barriers to change. The GRiP method aims to compare the findings of the audit, identify barriers and enablers to the utilization of evidence, and assist in developing implementation strategies to reduce the evidence-to-practice gap. The GRiP method can be undertaken in three stages: evaluating baseline audit findings, identifying barriers and enablers to evidence utilization, and developing and implementing strategies for change. | Evaluate baseline audit findings. Present findings to stakeholders, working groups, clinical staff. | HCW and patient interviews conducted to understand barriers and enablers to PADL. That and the wider literature have been used to develop this PADL complex intervention. | complete |  |
|  | Identify barriers and enablers to evidence implementation. Develop strategies for practice change. Use Geerligs SR findings to frame/categorise barriers/enablers from the local audit: system, staff, intervention. Use in-depth interviews, surveys, focus groups to identify barriers/enablers | One-to-one patient interviews will be undertaken to identify barriers and enablers to agreeing to PADL. These findings will be used to further optimise PADL. | nil |  |
|  | Developing strategies for change. Target strategies to overcome barriers using the GRiP approach | One-to-one HCW interviews (nurses, doctors, pharmacists) will be undertaken to identify barriers and enablers to delivering PADL. These findings will be used to further optimise PADL. | nil |  |
| **Phase 6: re-assess practice** Evaluation is a key component of implementing evidence into practice as it provides a systematic method to collect “information about the activities, characteristics, and outcomes of programs, services, policy, or processes, in order to make judgments about the program/process, improve effectiveness, and/or inform decisions about future development” | Evaluation of processes as per pre-implementation evaluation process to ensure comparable results and data | One-to-one HCW interviews (nurses, doctors, pharmacists) will be undertaken in order to identify barriers and enablers to delivering PADL. These findings will be used to further optimise PADL. | nil |  |
|  | Careful review of the differences between the baseline and follow-up results must be undertaken by the project team. | One-to-one patient interviews will be undertaken in order to identify barriers and enablers to agreeing to PADL. These findings will be used to further optimise PADL. | nil |  |
| **Phase 7: plan for sustainability** | Consideration should be given to who will be responsible for continuing to evaluate and improve the practice area and when it is feasible and appropriate to repeat a review of the practice area. | The antimicrobial pharmacists will be responsible for continuing to evaluate and improve the intervention. | Review plan at 6 months post implementation. |  |

**Reference** Porritt K, McArthur A, Lockwood C, Munn Z. JBI's approach to evidence implementation: a 7-phase process model to support and guide getting evidence into practice. JBI Evid Implement. 2023 Mar 1;21(1):3-13. doi: 10.1097/XEB.0000000000000361. PMID: 36545902.
