# Supplementary material for: Outcomes of a penicillin allergy de-labelling implementation intervention in a UK hospital: a prospective, single centre, type 2 hybrid effectiveness-implementation study evaluated using mixed methods
Source: Implement Sci Commun. 2026 Jun 11;7:157. doi: 10.1186/s43058-026-01008-8 (PMC13488184; doi:10.1186/s43058-026-01008-8)
Supplement: Supplementary file 3 [file 43058_2026_1008_MOESM4_ESM.docx]

| **Domains** | **Categories** | **Description** | **PADL intervention component** | **Comments & notes taken from the paper** |
| --- | --- | --- | --- | --- |
| System level barriers and facilitators | Environmental context | The physical, structural resources of the context, along with its processes and personal resources | PADL Champions / PADL leaders will champion and support the intervention. Ward pharmacists will support PADL, acting as prompts to doctors to PADL, and also initiating and delivering PADL themselves. | This is a challenge: Staff workload/competing demands/ tension juggling priorities, patient moves between wards. High staff turnover is cited as a barrier to implementation, but RCHT staff turnover is not too high. Delegation of some duties and support provided at the institutional level for implementation believed to be more sustainable facilitator. |
|  | Culture | The system culture, beliefs and behaviours in relation to change and staffing roles | There are good levels of trust between medical microbiology, antibiotic pharmacists and ward doctors, nurses, and pharmacists. Champion(s) will be selected to facilitate and motivate HCWs. Hospital approved PADL guidelines are published. There is congruence between PADL behaviours and staff roles. | System level commitment & change readiness. Need support from management regarding importance of change: organisational-level commitment to new processes. Use of champions or co-ordinators to facilitate motivation. Congruence between intervention requirements and staff roles are important. Culture barriers included the level of role flexibility and trust between different clinicians. |
|  | Communication processes | The processes of conveying information within the system, in terms of both online and in-person methods | The allergy focused history will be documented in its own dedicated notes within EPMA to allow easy access to the penA history for those wanting to review it. The results of the PADL will be communicated to the GP via two possible routes: EPMA note that populates the discharge letter, or a dedicated letter sent via email. Openness will be facilitated by the PADL champions and PADL leaders who will be visible and available for advice and for the reporting to of errors or issues. | This is about communication between different staff in disciplines where miscommunication could be a barrier to implementation - open and clear comms motivated staff to take on challenges and to feel safe about reporting errors or issues. |
|  | External requirements | Any external pressures or expectations that impact on the deliverables of the system | Guidelines on PADL as a a standard of care provide some perceived obligation to deliver PADL and delivery PADL as a hospital standard provides some motivation for some clinicians. Audit and feedback (SPC run charts) and reductions in broad spectrum antibiotics because of PADL will demonstrate the impact of PADL. Case studies where PADL had significant impact on improving patient care will be shared. There are no external pressures to PADL but there is WHO, DoH, BSACI, RPS *et al.* support for PADL. | Audits, external pressures such as pending audits, accreditation, assessments by external body were strong influences of change. Perception of external obligations was considered a source of motivation. Implementation as part of hospital policy or standards is a motivator. if intervention perceived as contributor to better institutional outcomes. |
| Staff | Staff commitment and attitudes | The micro-level beliefs, attitudes, and behaviours toward change in general, and the intervention specifically. | There is evidence of staff commitment from a wide-reaching survey from 2017 (acknowledgment that something needs to be done about incorrect penA). There is support from consultants in medical specialties evidenced by letters of support for RIPAL. The WP2 interviews (although selection bias an issue) demonstrate commitment and motivation. RIPAL-2 shows motivation to PADL. Belief in the intervention is there, but not strong. Instillation of stronger belief in PADL might be required amongst doctors, nurses, pharmacists. Sharing case studies where PADL has had a positive impact on patients might contribute to further motivating staff to PADL. No intention to force the intervention on HCWs, more that HCWs are given opportunity to participate in PADL. A sense of ownership may be instilled through a non-forced approach and discussing case studies with specialties and inviting feedback of the PADL intervention will engage HCWs in the process. Identifying PADL champions within the medical workforce will also instil a sense of engagement. | staff commitment and motivation for carrying out the intervention. Lack of belief in the intervention barrier. Change readiness levels influenced commitment to the intervention. Must not risk clinicians losing control of their role or feeling they are forced to make changes. A sense of ownership and a belief in the process are key facilitators. These were more likely if staff felt engaged in the process of implementation. |
|  | Understanding / awareness | Understanding of the aims and methodology of the intervention | Education and training will ensure aims and process is well understood. Ensure longer term positive outcomes of PADL are explained and that getting it right now will reduce workload in the future. Presenting PADL cases studies will highlight the potential benefits of PADL. | Staff knowledge of aims and process of the intervention key to ensuring effective implementation. Confusion or disregard of intervention processes could impact implementation. Where an intervention required additional work, it was important that staff understood that it would lead to longer term positive outcomes and reduction in overall burden. |
|  | Role identity | Beliefs and attitudes towards one’s work role and responsibilities | Qualitative interviews have shown that HCWs feel PADL aligns with their job role and hospital processes. HCWs view PADL is a shared responsibility. Presenting PADL cases studies will highlight the potential benefits of PADL and contribute to a sense of duty to deliver PADL. | Motivation to deliver an intervention waned when viewed as not part of job roles or where there was confusion regarding who’s role it was to deliver. Role responsibility was likely increased in situations where participants felt a sense of duty or obligation to the intervention. |
|  | Skills, ability, confidence | Staff sense of their capacity to carry out the tasks of the intervention, while managing the barriers posed by the target population and their work environment | Education and training will be provided, regularly and far reaching via multiple modes of delivery and in multiples locations. Time management was raised as a concern by HCWs in qualitative interviews but sharing the responsibility for PADL and doing it at more convenient times (daytime hours, not nurse hand over etc.) would facilitate delivery of PADL. PADL leads to a greater consistency of practice and so that needs to be highlighted in the training for HCWs. | Lack of confidence, or lack of ability was a significant barrier, especially if an area staff are not trained for. Ability to carry out the intervention was further impacted by stress and time management. Where an intervention led to greater consistency of practice then that was reported as a facilitator. |
| Intervention | Ease of integration | How well the intervention “fits” with the current system, resources and needs of the population and context, as well as its ability to adapt and respond when changes are needed | PADL fits with current healthcare system / flow and aligns with work processes and patient flow. PADL is viewed by HCWs as a simple process. PADL is acceptable to staff (with a little support). Communication of new allergy status to GPs is going to be a challenge as it is new but is flexible (multi-modes of delivery) and PADL is amenable to iterative change / improvement. However, there is a time burden and not all patients find PADL acceptable to them. The PADL intervention is a complex intervention with multiple components making it more challenge to deliver it in its entirety- this will require support from ward pharmacists and PADL champions. | Interventions that fitted the existing hospital system and ways of working were more likely to be reported as successful. Interventions that were flexible and iterative, such as those that engaged in ongoing tailoring and review were more successful. Intervention complexity often made integration more challenging. Where interventions required new operating systems, IT functionality and accessibility issues were commonly reported. Complexity also related to intervention design: interventions that involved multiple health professionals across a range of contexts increased the likelihood of delays and miscommunications. Similarly, interventions involving additional forms or screening tools created extra work for staff, and more errors in process were likely. This issue could often be targeted by simplifying forms and tools to make the process more user-friendly. Interventions that were perceived as simple and accessible were more likely to receive positive endorsement and greater engagement with the implementation process. Acceptability and suitability of an intervention to system, staff, and patient influenced how easily it was integrated. The cost and resources required by an intervention, in terms of work, time and stress, also influenced acceptability, and were often cited as reasons for withdrawing from, or having negative feelings toward, the implementation process. Finally, acceptability of the intervention to the patient was key to integration; staff encountered barriers where patients perceived that the intervention was not relevant. Staff highlighted the importance of considering this in the pre-implementation design phase. |
|  | Face validity/  evidence base | The extent to which the intervention is grounded in solid evidence regarding a known issue, and how effective it looks to be in terms of meeting its aims | The evidence for PADL will be communicated in education sessions rolled out to healthcare workers. SPC run charts will show the PADL activity and audit data will demonstrate safety and the impact on broad spectrum antibiotic use. | Many participants expressed concerns about the evidence base of interventions, and this was frequently cited as a barrier to implementation. Communicating and making the evidence accessible to staff in was considered a key facilitator as lack of awareness of the evidence was commonly reported. When participants felt confident about the evidence and the intervention rationale, this increased motivation to support the implementation overall. |
|  | Safety/ legal/ ethical concerns | How well an intervention addresses important issues of safety and legality to protect staff and patients | Education sessions will communicate the safety of PADL and the potential for PADL to improve patient care. SPC run charts will be used to evidence safety in our patient cohort. | Many participants raised concerns about intervention safety, particularly where change of care was required. Participants raised this as a barrier when they were asked to deliver information they did not agree with. Conversely, an intervention perceived as leading to potentially decreased risks and improved care was seen as a facilitator. Ethical issues concerning patient well-being and patient confidentiality were sometimes raised. For example, when interventions required shared platforms, participants noted that confidentiality relating to user privacy needed to be considered and that patient awareness of the shared platform could influence information disclosed. Concerns regarding legality and fear of litigation were also commonly cited barriers when interventions called for changes in roles and responsibilities. Concerns about safety meant that staff were less likely to endorse or fully participate in the implementation. |
|  | Supportive components | The components of the intervention which work to support and facilitate the changes necessary | We will train as many as possible but also enable those trained to train others such that training is cascaded. Training will be delivered in the HCW's environment, i.e. in already established training sessions or on the ward. Sustained awareness campaigns will be required to support continued engagement with PADL. Continuous data collection will enable demonstrable patient safety for our local cohort of patients - will motivate staff. Staff will be engaged with the intervention through dialogue with staff along the way. Initially, pre-intervention, there have been surveys, focus groups and one-to-one interviews with staff. | Training, awareness raising, audit/feedback, and engagement with end users could all serve as barriers or facilitators. Lack of training and awareness of intervention processes was seen as a key barrier. Repeated training and awareness campaigns were seen as necessary to reinforce new processes and behavioural patterns, although access and time to attend training, along with availability of professional support, were common challenges. These awareness-raising activities were perceived as most useful when they highlighted the evidence and need for the intervention, as well as the likely benefits to staff and patients. The importance of regular audit, such as real-time monitoring of admissions to ensure fidelity, was also reported as helpful to the implementation success. These strategies were also associated with improved motivation and demonstrated the utility of the intervention. Finally, participants highlighted the importance of engaging with the intervention end users (i.e., themselves and their colleagues) to facilitate the process of implementation in a way that was acceptable, appropriate, and sustainable. Studies which had adopted models of iterative implementation, such as participatory action research, reported greater engagement from end users. |

**Reference** Geerligs L, Rankin NM, Shepherd HL, Butow P. Hospital-based interventions: a systematic review of staff-reported barriers and facilitators to implementation processes. Implement Sci. 2018 Feb 23;13(1):36. doi: 10.1186/s13012-018-0726-9. PMID: 29475440; PMCID: PMC5824580.
