# Supplementary material for: Outcomes of a penicillin allergy de-labelling implementation intervention in a UK hospital: a prospective, single centre, type 2 hybrid effectiveness-implementation study evaluated using mixed methods
Source: Implement Sci Commun. 2026 Jun 11;7:157. doi: 10.1186/s43058-026-01008-8 (PMC13488184; doi:10.1186/s43058-026-01008-8)
Supplement: Supplementary file 4 [file 43058_2026_1008_MOESM5_ESM.docx]

**Appendix A**

**Penicillin Allergy De-Labelling in Adult Inpatients Clinical Guideline**

**V1.1**

**June 2024**

**Summary**

**Aim/Purpose of this Guideline**

The purpose of this guideline is to support safe penicillin allergy de-labelling and optimise antibiotic treatment.

Removing inappropriate penicillin allergy records reduces unnecessary second and third-line antibiotics and reduces broad spectrum antibiotic usage in hospitalised patients.

Most patients with a penicillin allergy label (>95%) do not have a true penicillin allergy and can take penicillin safely.

This guideline is designed to support and facilitate penicillin allergy risk assessment, to assess whether patients can be de-labelled safely.

This version supersedes any previous versions of this document.

**Data Protection Act 2018 (UK General Data Protection Regulation – GDPR) Legislation**

The Trust has a duty under the Data Protection Act 2018 and UK General Data Protection Regulations 2016/679 to ensure that there is a valid legal basis to process personal and sensitive data. The legal basis for processing must be identified and documented before the processing begins. In many cases we may need consent; this must be explicit, informed, and documented. We cannot rely on opt out, it must be opt in.

Data Protection Act 2018 and UK General Data Protection Regulations 2016/679 is applicable to all staff; this includes those working as contractors and providers of services.

For more information about your obligations under the Data Protection Act 2018 and UK General Data Protection Regulations 2016/679 please see the Information Use Framework Policy or contact the Information Governance Team

Royal Cornwall Hospital Trust	[rch-tr.infogov@nhs.net](mailto:rch-tr.infogov@nhs.net)

**The Guidance**

**Allergy assessment**

Adult patients (>18 years of age) admitted to hospital with a penicillin allergy record preventing the use of a penicillin antibiotic and have a low-risk allergy record are eligible for consideration of Penicillin Allergy De-labelling (PADL).

A structured penicillin allergy history should be obtained from the patient, and other sources of information, by a doctor or pharmacist using the questions shown in Appendix 3.

From the responses to these questions, use the validated allergy assessment tool shown in Appendix 4 to categorise the patient into one of the three allergy risk categories: ‘Low risk appropriate for direct de-label on history alone’, ‘Low risk eligible for direct oral challenge (DOC) test’ or ‘High risk’. NB the assessment tool needs to be viewed in colour. It is available via Microguide, accessed in the mobile phone app or via the documents library.

Cut and paste the questions and the answers given into a ‘Pharmaceutical Care Plan’ note in EPMA with a title that reflects the outcome of the allergy risk assessment. Choose one of the following titles:

“PADL HIGH” – high risk allergy history as per decision tool.

“PADL LOW DDL” - low risk allergy history as per decision tool, eligible for direct de-label (removal of penicillin allergy record after allergy history reconciliation).

“PADL LOW DOC” - low risk allergy history as per decision tool, eligible for Direct Oral Challenge.

“PADL unable to obtain history (revisit)” - if the reason for non-obtaining history is likely to resolve during inpatient stay.

“PADL unable to obtain history”- if the reason for non-obtaining history is likely permanent.

High-risk patients are excluded at this stage with appropriate sign posting and referred for specialist allergy assessment via the outpatient allergy services in Plymouth, (see section 2.4), where appropriate.

If the patient is assessed as low risk, then follow the process in the appropriate de-label pathway below (either DDL or DOC). If there is evidence that the patient has received a penicillin since the index reaction use Appendix 5 to determine appropriateness for DDL and follow DDL process below.

**Direct de-label (DDL: de-label on history alone after allergy history reconciliation)**

Counsel the patient on the risks and benefits of penicillin allergy records using the Information sheet in Appendix 6.

With the patient’s consent, remove the incorrect penicillin allergy record from EPMA entirely or move it to the intolerance section of EPMA, if more appropriate to do so.

It is good practice to add a note to EPMA stating that the patient’s allergy history has been removed, stating the history given by the patient and why it was removed. This will potentially help prevent re-labelling of the penicillin allergy during future episodes of care.

Provide the patient with the leaflet in Appendix 7.

If the GP records show a penicillin allergy record, then notify the GP of the amended allergy status either by emailing the letter in Appendix 8 with the patient’s details (preferred option) or adding a EPMA note ‘note to appear in GP letter’ stating the history given by the patients and the reason for de-label e.g. “Patient [name] reports an intolerance to penicillin (nausea) so we have removed this from their drug allergy box and prescribed amoxicillin for their CAP, which was well tolerated.”

**Direct Oral Challenge (DOC)**

Counsel the patient on the risks and benefits of penicillin allergy records and the risks and benefits of DOC using the Information sheet in Appendix 6.

Consent the patient to an oral challenge test (single oral dose of index penicillin or amoxicillin if index penicillin unknown). Verbal patient consent needs will be sought prior to prescribing the dose of penicillin.

The responsible clinician for the patient must provide consent for their patient to have an oral challenge dose.

Check the patient does not meet any exclusion criteria for a direct oral challenge test (see list below).

Exclusion criteria:

- Pregnancy.
- Cognitive impairment.
- Where a collateral history cannot be obtained.
- History of drug-associated anaphylaxis or oedema.
- History of severe cutaneous adverse reactions.
- History of acute kidney injury or severe liver impairment associated with antibiotic therapy.
- Haemodynamic instability (defined as BP or HR outside normal range).
- History of brittle / or severe asthma or has (had a course of steroids in the past 3 months for asthma).
- Unstable coronary artery disease.
- Uncontrolled heart failure.
- Requiring oxygen to maintain oxygen saturations above 92% (relative contraindication, to be assessed in the context of the acuity of illness).
- Antihistamines if cannot be withheld on day of testing.

Discuss any uncertainties you have about challenge testing with either a medical microbiologist, infectious diseases consultant or antimicrobial stewardship pharmacist.

Follow the DOC protocol in Appendix 9.

Oral challenge testing must only be undertaken in an inpatient environment on the ward with access to anaphylaxis drugs and expertise in anaphylaxis management. Patients must be closely observed and must not leave the ward during testing.

Ward nursing team or challenge test healthcare worker (pharmacist or doctor) to perform basic observations as per DOC protocol (Appendix 9) before, and after the DOC.

If there is no evidence of a reaction one hour post direct oral challenge, then the allergy records should be amended, with the patient’s consent, and the GP notified of the removal of their penicillin allergy label.

It is good practice to add a note to EPMA stating that the patient’s allergy history has been removed stating the history given by the patient and why it was removed. This will potentially help prevent re-labelling of the penicillin allergy during future episodes of care.

If the GP records show a penicillin allergy record, then notify the GP of the amended allergy status either by emailing the letter in Appendix 8 with the patients details or adding a ‘note to appear in GP letter’ EPMA note stating the history given by the patients and the reason for de-label e.g. “[Patient name] reports a penicillin allergy but upon questioning patient, the patient reports a benign skin reaction with penicillin >10 years ago. After tolerating a test dose of amoxicillin, we have removed this allergy record. The patient tolerated a 5-day course of amoxicillin during current inpatient stay”.

Inform the patient of their negative test result and counsel the patient of the 1-2% risk of a delayed benign skin reaction because of the penicillin exposure. Give the patient information leaflet in Appendix 7.

In the unlikely event the patient has a positive reaction (examples of potential manifestations provided in Appendix 10) to the oral dose of penicillin then give the patient the information in Appendix 11.

Notify the GP of the confirmed positive allergy status using the letter in Appendix 12 or add a ‘note to appear in discharge letter’ EPMA note stating the history given by the patients and the symptoms experienced that are in keeping with a positive allergic reaction e.g., “[Patient name] reports a penicillin allergy but upon questioning patient, the patient reports a benign skin reaction with penicillin >10 years ago. After exposure to amoxicillin [patient name] experienced a maculo-papular rash after 5 days. The patient is to retain their penicillin allergy status.”

**Referral to allergy services (high risk)**

Patients meeting criteria for specialist allergy assessment can be referred by their GP to outpatient allergy services in Plymouth, in accordance with NICE Drug Allergy Guideline (CG183) and local referral guidelines (see link below). These include patients with a history of:

Suspected anaphylaxis.

Non-immediate non-severe cutaneous reaction where that class of drug is considered essential to management.

A severe non-immediate cutaneous reaction.

Beta lactam allergy when (a) beta lactams are considered essential for management (b) there is likely to be frequent need for beta-lactam antibiotics in the future (e.g., recurrent bacterial infections or immune deficiency) (c) there is suspected allergy to at least one other class of antibiotics in addition to beta lactams.

Local referral document here: <https://rms.cornwall.nhs.uk/rms/primary_care_clinical_referral_criteria/allergy>

**Documentation of confirmed allergy status**

If allergy confirmed to be high risk, then update allergy record on EPMA and ensure the allergy record documented on EPMA reflects the patient history given. If it does not, then update EPMA.

Ensure the GP record reflects the allergy status with correct allergy history documented.

If it does not, then add a note for GP (“note to appear in discharge summary” via EPMA).

**Task specific responsibilities**

The ward teams (either a medication optimisation pharmacy technician, ward pharmacist, antibiotic stewardship pharmacy technician, antibiotic pharmacist, or doctor) will take a structured penicillin allergy history using the set allergy history questions in this document and document the outcome in a Pharmaceutical Care Plan (PCP) note.

Ward pharmacists, antibiotic pharmacists and doctors can remove penicillin allergy record via direct de-label if they feel competent to do so. (Please see suggested education resources in section 2.8).

Ward pharmacists, antibiotic pharmacists and doctors can decide whether a patient is eligible for a direct oral challenge test using the risk stratification tool in this document, if they deem themselves competent to do so. (Please see suggested education resources in section 2.8).

Prescribing a direct oral challenge dose can be done by a non-medical prescribing pharmacist or doctor with permission from the patient’s responsible clinician.

The post-dose observations should ideally be carried out by a trained nurse but can be undertaken by others including healthcare assistants, medical and nursing students, ward pharmacists and ward doctors.

**Testing environment**

Direct oral challenge testing must be done in a ward with access to trained medical and nursing staff and with access to the resuscitation team in the rare event the patient has a severe reaction.

**Education resources on penicillin allergy de-labelling for non-allergists**

In the electronic staff record (ESR) select ‘learning certification’ and search ‘penicillin’ for education module on direct de-label on history alone.

British Society of Antimicrobial Chemotherapy have a Massive Open Online Module on penicillin allergy (6 hours of learning over three weeks).

Journal Article ‘Evaluation and Management of Penicillin Allergy A Review’ by Erica S. Shenoy, Theresa Rowe, Kimberly G. Blumenthal. JAMA. 2019;321(2):188-199.doi:10.1001/jama.2018.19283.

**Monitoring compliance and effectiveness**

| **Information Category** | **Detail of process and methodology for monitoring compliance** |
| --- | --- |
| **Element to be monitored** | The number of patients risk assessed, the appropriateness of the allergy risk assessment, the numbers of patients meeting high risk and low risk criteria and the numbers safely de-labelled by both DDL and DOC. |
| **Lead** | Neil Powell and the antimicrobial stewardship team. |
| **Tool** | Monitored as part of an NIHR funded doctoral fellowship initially (first 6-12 months), then monitored as part of antimicrobial stewardship activity in the hospital. |
| **Frequency** | Ongoing audit, reported monthly. |
| **Reporting arrangements** | Antimicrobial Stewardship Management Committee (ASMC).  Medicines Practice Committee (MPC). |
| **Acting on recommendations and Lead(s)** | The ASMC will lead on subsequent recommendations made by the MPC. |
| **Change in practice and lessons to be shared** | Required changes to practice will be identified and actioned within a month. A lead member of the team will be identified to take each change forward where appropriate. Lessons will be shared with all the relevant stakeholders. |

**Equality and Diversity**

This document complies with the Royal Cornwall Hospitals NHS Trust service Equality and Diversity statement which can be found in the [Equality Diversity And Inclusion Policy](https://doclibrary-rcht.cornwall.nhs.uk/DocumentsLibrary/RoyalCornwallHospitalsTrust/HumanResources/EqualityDiversityAndInclusionPolicy.pdf) or the [Equality and Diversity website](http://intranet-rcht.cornwall.nhs.uk/shelf/equality-and-diversity/).

Equality Impact Assessment

The Initial Equality Impact Assessment Screening Form is at Appendix 2.

**Appendix 1. Governance Information**

| **Information Category** | **Detailed Information** |
| --- | --- |
| **Document Title:** | Penicillin Allergy De-Labelling in Adult Inpatients Clinical Guideline V1.1 |
| **This document replaces (exact title of previous version):** | New Document |
| **Date Issued/Approved:** | 27 October 2023 |
| **Date Valid From:** | February 2023 |
| **Date Valid To:** | February 2023 |
| **Directorate / Department responsible (author/owner):** | Neil Powell, Consultant Antimicrobial Pharmacist. |
| **Contact details:** | 01872 252593  [rcht_penicillin@nhs.net](mailto:rcht_penicillin@nhs.net) |
| **Brief summary of contents:** | Penicillin allergy de-labelling guideline. |
| **Suggested Keywords:** | Penicillin, penicillin allergy, de-label, de-labelling. |
| **Target Audience:** | **RCHT:** Yes  **CFT:** No  **CIOS ICB:** No |
| **Executive Director responsible for Policy:** | Chief Medical Officer |
| **Approval route for consultation and ratification:** | Medicine Practice Committee (MPC) |
| **Manager confirming approval processes:** | Richard Andrzejuk |
| **Name of Governance Lead confirming consultation and ratification:** | Kevin Wright |
| **Links to key external standards:** | Start smart then focus: antimicrobial stewardship toolkit for inpatient care settings. Updated 12 September 2023. <https://www.gov.uk/government/publications/antimicrobial-stewardship-start-smart-then-focus/start-smart-then-focus-antimicrobial-stewardship-toolkit-for-inpatient-care-settings> |
| **Related Documents:** | BSACI guideline for the set-up of penicillin allergy de-labelling services by non-allergists working in a hospital setting https://doi.org/10.1111/cea.14217. |
| **Training Need Identified?** | Yes. Education and training will be delivered by the antimicrobial stewardship pharmacists to ward pharmacists, doctors, and nurses.  Signposting to education resources given in the guideline above see “2.8 Education resources on penicillin allergy de-labelling for non-allergists”. |
| **Publication Location (refer to Policy on Policies – Approvals and Ratification):** | Internet and Intranet |
| **Document Library Folder/Sub Folder:** | Clinical / Pharmacy |

**Version Control Table**

| **Date** | **Version Number** | **Summary of Changes** | **Changes Made by** |
| --- | --- | --- | --- |
| October 2023 | V1.0 | Initial issue | Neil Powell Consultant Antimicrobial Pharmacist |

**All or part of this document can be released under the Freedom of Information Act 2000.**

**All Policies, Strategies and Operating Procedures, including Business Plans, are to be kept for the lifetime of the organisation plus 6 years.**

**This document is only valid on the day of printing.**

**Controlled Document.**

This document has been created following the Royal Cornwall Hospitals NHS Trust [The Policy on Policies (Development and Management of Knowledge Procedural and Web Documents Policy)](https://doclibrary-rcht.cornwall.nhs.uk/DocumentsLibrary/RoyalCornwallHospitalsTrust/HealthInformatics/CorporateAndHealthRecords/ThePolicyOnPoliciesDevelopmentAndManagementOfKnowledgeProceduralAndWebDocumentsPolicy.pdf). It should not be altered in any way without the express permission of the author or their Line Manager.

**Appendix 2. Equality Impact Assessment**

**Section 1: Equality Impact Assessment (EIA) Form**

The EIA process allows the Trust to identify where a policy or service may have a negative impact on an individual or particular group of people.

For guidance please refer to the Equality Impact Assessment Policy (available from the document library) or contact the Equality, Diversity, and Inclusion Team [rcht.inclusion@nhs.net](mailto:rcht.inclusion@nhs.net)

| **Information Category** | **Detailed Information** |
| --- | --- |
| **Name of the strategy / policy / proposal / service function to be assessed:** | Penicillin Allergy De-Labelling in Adult Inpatients Clinical Guideline V1.1 |
| **Directorate and service area:** | Pharmacy, Clinical Support. |
| **Is this a new or existing Policy?** | New |
| **Name of individual completing EIA** (Should be completed by an individual with a good understanding of the Service/Policy): | Neil Powell Consultant Antimicrobial Pharmacist |
| **Contact details:** | 01872 252593 |

| **Information Category** | **Detailed Information** |
| --- | --- |
| 1. **Policy Aim - Who is the Policy aimed at?**   (The Policy is the Strategy, Policy, Proposal or Service Change to be assessed) | Doctors, nurses, medicines optimization pharmacy technicians and pharmacists. |
| 2. **Policy Objectives** | To remove incorrect penicillin allergy records from patient medical notes. |
| 3. **Policy Intended Outcomes** | More patients receiving first line antimicrobial agents. |
| 4. **How will you measure each outcome?** | Audit. |
| 5. **Who is intended to benefit from the policy?** | Patients, healthcare systems, wider society. |
| **6a. Who did you consult with?**  (Please select Yes or No for each category) | - Workforce: Yes - Patients/ visitors: Yes - Local groups/ system partners: No - External organisations: No - Other: No |
| **6b. Please list the individuals/groups who have been consulted about this policy.** | **Please record specific names of individuals/ groups:**  Medical consultants, intensive care consultants.  Medicine Practice Committee (MPC). |
| **6c. What was the outcome of the consultation?** | Positive, agreed. |
| **6d. Have you used any of the following to assist your assessment?** | **National or local statistics, audits, activity reports, process maps, complaints, staff, or patient surveys:**  Staff |

**7. The Impact**

Following consultation with key groups, has a negative impact been identified for any protected characteristic? Please note that a rationale is required for each one.

Where a negative impact is identified without rationale, the key groups will need to be consulted again.

| **Protected Characteristic** | **(Yes or No)** | **Rationale** |
| --- | --- | --- |
| **Age** | No |  |
| **Sex** (male or female) | No |  |
| **Gender reassignment** (Transgender, non-binary, gender fluid etc.) | No |  |
| **Race** | No |  |
| **Disability** (e.g. physical or cognitive impairment, mental health, long term conditions etc.) | No |  |
| **Religion or belief** | No |  |
| **Marriage and civil partnership** | No |  |
| **Pregnancy and maternity** | No |  |
| **Sexual orientation** (e.g. gay, straight, bisexual, lesbian etc.) | No |  |

**A robust rationale must be in place for all protected characteristics. If a negative impact has been identified, please complete section 2. If no negative impact has been identified and if this is not a major service change, you can end the assessment here.**

I am confident that section 2 of this EIA does not need completing as there are no highlighted risks of negative impact occurring because of this policy.

Name of person confirming result of initial impact assessment: Neil Powell Consultant Antimicrobial Pharmacist.

**If a negative impact has been identified above OR this is a major service change, you will need to complete section 2 of the EIA form available here:**
[Section 2. Full Equality Analysis](http://doclibrary-rcht-intranet.cornwall.nhs.uk/DocumentsLibrary/RoyalCornwallHospitalsTrust/ChiefExecutive/Templates/Section2FullEqualityAnalysis.docx)

**Appendix 3. Penicillin allergy assessment questions**

The answers to the questions below will be used with the decision support tool below to decide whether the patient is de-labelled on history alone, is offered an oral challenge test or whether no intervention is able to be offered. Those meeting criteria for allergist referral will be considered for allergist referral if they meet the local referral criteria (below). Cut and paste the questions and the text below into an EPMA Pharmaceutical care plan note with ‘PADL’ as the title.

1. **Which penicillin did you react to?**
2. **Do you remember the details of the reaction?**
3. **How many hours after having your first dose of the antibiotic did the reaction occur?**
4. **How many years ago did the reaction occur?**
5. **How was the reaction managed? What was the outcome?**
6. **Which other antibiotics have you tolerated post reaction?**

Penicillin allergy history taken by: (name and job role)

Date

Risk assessment categorisation:

Proposed action:

Risk assessment person (name and job role)

Date

**Appendix 4. Validated Allergy Assessment Tool**

**Appendix 5. Managing patients who have tolerated a penicillin since the index reaction**

| **Scenario** | **Recommendation** | **Explanation** |
| --- | --- | --- |
| A history consistent with an IgE mediated reaction to a known penicillin and has tolerated the same penicillin or amoxicillin since the index reaction. | De-label on history alone. | Tolerating the index penicillin rules out IgE mediated reactions. Tolerating amoxicillin rules out IgE reaction to the beta-lactam ring a the most common R1 side chain allergen. Amoxicillin is the penicillin used to definitively rule out IgE allergic reactions during formal allergy testing. |
| A history consistent with an IgE mediated reaction to a known penicillin or unknown penicillin and has tolerated a penicillin antibiotic since but it was not the index penicillin or amoxicillin. | Cannot de-label based on the fact they have since tolerated a penicillin. | IgE mediated reactions can be due to the beta-lactam ring or the R1 side chain. Selective reactivity can occur to amoxicillin, piperacillin-tazobactam and flucloxacillin R1 side chains. American studies suggest selective IgE mediated allergy to amoxicillin is very rare whereas European studies suggest it may be between 25-50% of patients with positive skin tests have selective allergy to amoxicillin. |
| A history consistent with a low-risk allergy history that meets criteria for a DOC test but has tolerated any penicillin / unknown penicillin since the index reaction. | De-label on history alone. | Low risk allergy histories that meet criteria for DOC are not likely to re-react to penicillin when challenged. Ruling out beta-lactam ring sensitivity is sufficient in these patients. |
| An allergy history consistent with a severe delayed reaction (Gell and Coombs types 2-4) and includes DILI, cytopenia, AIN, AGEP, DRESS, SJS/TEN and tolerated any penicillin or unknown penicillin since index reaction. | Avoid all beta-lactams | High risk allergy group and little is understood about the best method to de-label these patients. Prior tolerance does not rule out severe delayed reactions on subsequent exposure. |

**Appendix 6.** [**Penicillin allergy de-labelling (RCHT 2060)**](https://doclibrary-rcht.cornwall.nhs.uk/DocumentsLibrary/RoyalCornwallHospitalsTrust/PatientInformation/Pharmacy/RCHT2060PenicillinAllergyDelabelling.pdf)

**Appendix 7.** [**You do NOT have an allergy to penicillin (RCHT 2061)**](https://doclibrary-rcht.cornwall.nhs.uk/DocumentsLibrary/RoyalCornwallHospitalsTrust/PatientInformation/Pharmacy/RCHT2061YouDoNotHaveAnAllergyToPenicillin.pdf)

**Appendix 8. Penicillin Allergy Record – Remove Penicillin Allergy Label**

Date [today]

Patient name [xxxxxx]

Patient NHS no [xxxxxxx]

Dear Dr,

Your patient [John Smith] underwent assessment of their penicillin allergy label during a recent hospital attendance.

After review of their history, and discussion of the risk and benefits, we removed this allergy record from our hospital medical notes.

Patient gave a history as follows: [e.g. benign skin reaction in child hood / nausea].

Subsequent exposure to a penicillin antibiotic: [amoxicillin was given for 5 days / we did not expose the patient to penicillin on this admission].

Patient reports side effects: [the course was tolerated / we did not expose the patient to penicillin on this admission].

Based on this we can advise you that **there is no evidence to now support a “penicillin allergy” label.** We request that you remove this allergy label from your patient’s medical record and record details of the allergy test (antibiotic and date). This information should be shared with other healthcare providers within your Practice and their regular community pharmacy and general dental practitioner if possible.

The risk of allergic reaction to penicillin in a de-labelled patient is the same as that of the general population and **they can therefore receive penicillin antibiotics**. This is important for management of any future infections as penicillins are often recommended as first line therapy due to their effectiveness and lower risk of driving antimicrobial resistance.

**Your patient has also been informed that they can safely take penicillin-based antibiotics in future** and a copy of the information which they received after the test is included with this letter.

If you have any queries regarding the test process or outcome, please do not hesitate to contact me.

Thanks and best wishes.

Name and Grade

Enquiries contact:

Acknowledgments: letter adapted from Health Protection Scotland Scottish Antimicrobial Prescribing Group Penicillin allergy de-labelling intervention.

**Appendix 9. Direct oral challenge test procedure**

**Preparation**

1. Review the exclusion criteria for direct drug provocation testing.
2. Select the antibiotic to be used. In most cases this should be the penicillin antibiotic to which the patient had the adverse reaction. If the antibiotic is unknown, then amoxicillin is an appropriate choice.
3. Discuss the plan for an oral penicillin challenge with the patient and give them the patient information sheet.
4. Record in the case notes that consent has been obtained.

**Procedure for the direct drug provocation test**

1. Measure the patient’s observations (HR, BP, oxygen saturations, RR). If the patient has asthma, then measure peak expiratory flow rate (PEFR).
2. Prescribe and administer the antibiotic. Patient and observer to remain within the clinical area for 60 minutes.
3. Antibiotics should be administered as a single therapeutic oral dose: e.g. Amoxicillin 500mg.
4. Inform the patient to notify you immediately if they experience any adverse symptoms.
5. Ask the patient to report any symptoms and measure the patient’s observations (BP, pulse, Sp02, and PEFR if indicated) and at regular intervals e.g., at, 20 minutes, 40 minutes and 60 minutes and document.
6. Record any symptoms that the patient experiences.
7. If the patient reports any of the symptoms of a positive test (listed below) or they have a rising NEWS score, then the patient should be reviewed immediately by an appropriate senior member of staff.

**Post-procedure care**

1. Interpret the oral challenge with the following outcomes:
   a. Negative Test - no symptoms reported during the period of observation and patient’s NEWS score does not rise. Patient experiences isolated nausea or isolated itch without any of the other features of a positive test.
   b. Equivocal test - if there is doubt about the interpretation of the test then it should be discussed with a senior clinician and discussion with the local allergy service should be considered.
   c. Positive Test - patient experiences any of the following: itchy rash, breathing difficulties, facial swelling, hypotension, collapse, tongue swelling.
2. If the challenge is negative give the patient the patient information leaflet, record in the discharge letter and ask the patient’s GP to amend their allergy status on the practice records.
3. In case of late phase response, the patient must be instructed to call 111 or visit their local Emergency Department should they develop symptoms of dyspnoea, wheezing, dizziness, or severe pruritus.
4. If the challenge outcome is positive written and electronic record must clearly state this. The patient should be provided with the information leaflet and the GP informed of this outcome.

**Appendix 10. Signs and symptoms of potential allergy**

Signs and Symptoms of allergic reactions in various target organs. (Used with permission from the ALABAMA study Team).

**Signs and symptoms of potential allergy:**

Skin:	Urticaria/Angioedema.

Flushing.

Erythematous pruritic rash.

Atopic dermatitis.

Gastro-intestinal tract:	Pruritis and /or swelling of the lips, tongue, or oral mucosa.

Nausea.

Abdominal cramping or colic.

Vomiting or reflux.

Diarrhoea.

Respiratory tract:	Nasal congestion.

Rhinorrhoea.

Pruritis/sneezing.

Laryngeal oedema, staccato cough and/or dysphonia.

Wheezing/ repetitive cough.

Dyspnoea.

Cardiovascular:	Hypotension/shock.

Dizziness.

**Appendix 11.** [**Positive penicillin allergy testing result (RCHT 2062)**](https://doclibrary-rcht.cornwall.nhs.uk/DocumentsLibrary/RoyalCornwallHospitalsTrust/PatientInformation/Pharmacy/RCHT2062PositivePenicillinAllergyTestingResult.pdf)

**Appendix 12. Penicillin Allergy Test – Reaction Confirmed**

Dear Dr XXXXXXXXXXXXXX,

Your patient XXXXXXXXX XXXXXXXXXXX underwent assessment of their penicillin allergy label during a recent hospital attendance.

After review of their history, and discussion of the risk and benefits, a supervised oral challenge was performed. A dose of 500mg of **amoxicillin / flucloxacillin** was administered on **[date]**.

**There was evidence of an allergic reaction,** as detailed below:

xx

Please record this description and date of observation in their medical record as confirmation of their allergy status. Please also ensure their allergy status is clearly documented in all future correspondence/records.

The patient has been informed that they **should not take penicillin-based antibiotics** in future. Examples of these antibiotics include phenoxymethylpenicillin (penicillin V), amoxicillin, flucloxacillin and co-amoxiclav (Augmentin). A copy of the information which your patient has received after the test is included with this letter.

Thanks and best wishes

Name/Grade

Enquiries/Contact:

Acknowledgments: letter adapted from Health Protection Scotland Scottish Antimicrobial Prescribing Group Penicillin allergy de-labelling intervention

**Penicillin allergy de-labelling patient pathway**

A penA (we also include patients with a history of penicillin sensitivity) focused history was taken and risk assessed for likelihood of a serious allergy to penicillin, using the tool developed by Devchand *et al.* Patients categorised as ‘low risk’ were counselled about the risks and benefits of PADL before being offered direct de-labelling (based on history alone, DDL) or a direct oral challenge test (DOCT). If they consented to DOCT, permission was sought from the senior responsible clinician prior to prescription of the DOCT dose. And observations undertaken by a ward nurse. If no symptoms were reported within one hour, then the patient was counselled on their de-labelled status and provided with verbal and written counselling/information on the risks of future reactions (within next 5 days and upon future exposure to penicillin) and the GP informed of new penA status with a request to update their GP records via the discharge letter or dedicated PADL letter. Ward pharmacists were encouraged to risk assess patients’ documented penA histories and determine their risk category using the decision support tool, determine patient eligibility for DDL or DOCT, and indicate the risk category in the title of the pharmaceutical care plan PADL note, e.g. ‘PADL LOW DDL’. If the ward pharmacist was confident to de-label, then they were encouraged to do so. If not, the PADL note acted as a flag on a purpose build local intranet page behind NHS firewalls and reviewed daily by the AMS pharmacy team.

**Implementation phases**

*Pre-implementation phase:*

The core implementation team were two antimicrobial pharmacists working at the hospital (NP and DH). Pre-implementation phase plans included hospital management approval of the PADL guidelines, MOPTs received education on taking and documenting a penA focused history, ward pharmacists completed part 1 of the training (pre-recoded slide set on how to take and document a penA history and the PADL process) (see training material in appendix). Foundation year 1 and 2 doctors received part 1 training at a weekly face-to-face education meeting. All doctors and pharmacists who had completed part 1 were offered part 2 training (face to face case-based learning) (see training material in appendix). Infection specialists (one ID consultant and five medical microbiologists) were briefed by the PADL champion (NP) on the PADL process and requested to prompt penA history taking when giving advice, ward pharmacists and the AMS pharmacists for help with PADL, if required. A real time surveillance report was developed by a pharmacy data analyst which identified patients on medical and surgical wards with penicillin allergy or sensitivity records and an antibiotic prescription.

*Phase 1 (implementation: first 26 weeks)*

PADL champion (NP) attended specialty meetings to brief senior clinicians and nurses on the PADL intervention and to obtain their support for PADL. The chief pharmacist raised awareness of PADL at a whole of pharmacy departmental meeting, demonstrating senior pharmacy support.

Both PADL champions promoted both parts of PADL training at specialty and departmental meetings and during clinical ward duties and ensured uptake of part 1 training by eligible HCWs. AMS pharmacists championed PADL whilst delivering PADL in ward areas as part of their daily PADL ward round. Local data on numbers of patients de-labelled across the hospital and case studies were fed back to pharmacy clinical staff at least monthly and to medical staff when able at the Grand Round and newsletters emailed to clinical staff. PADL education was included in the annual consultant mandatory training, junior doctor training, new MOPT training, pharmacist and trainee pharmacist starters training. PADL data and case studies were communicated to specialties periodically.

*Phase 2 (sustainability): weeks 26 onwards*

AMS pharmacist ward round frequency reduced to once or twice weekly and to focus on patients with greatest likelihood to benefit from PADL, i.e. those where PADL would likely result in immediate optimisation of antibiotics, for example a patient with *Staphylococcus aureus* bacteria on teicoplanin when flucloxacillin would be the preferred antibiotic.

**Appendix B**

The mean number of patients with a penA record discharged from medical and surgical specialties per month between June 2024 and December 2024 was 342 (SD 24.32). The mean proportion of those exposed to any antibiotic during their inpatient stay was 55% (SD 1.57%).

B1

The mean number with a structured penA history documented on EPMA per month was 159 (SD 26.26). PADL note numbers were highest in July, August, September, October and November with an average of 174 / month over those four months and then dropped in December when the intervention moved to a sustainability phase on 20th December 2024 and AMS PADL ward round frequency was reduced.

Total penA history (with or without antibiotic prescribed) and PADL note added to EPMA

|  |  |
| --- | --- |
| June | 106 |
| July | 181 |
| August | 185 |
| September | 161 |
| October | 182 |
| November | 159 |
| December | 141 |

Graph B1 The number of patients with a structured penicillin allergy history (“PADL note”) each month.

***B2*** *Missed opportunities*

Of those 1283 penA patients without a structured penA history documented in EPMA (PADL note), 600 (47%) had had their medicines reconciled by a member of the pharmacy team and all patients had been seen by a ward doctor. The number of penA patients without a PADL note but had had their medications reconciled by pharmacy ranged between 51(32%) per month and 137(56%) per month.

Number of patients without a PADL note but with a pharmacy meds rec note

|  |  |
| --- | --- |
| June | 137 |
| July | 84 |
| August | 97 |
| September | 71 |
| October | 51 |
| November | 74 |
| December | 86 |

Graph B2 number of patients without a structured penicillin allergy history (“PADL note”) but who had had their medicines reconciled by a member of the pharmacy team.

Of 1323 penA patients prescribed any antibiotic 735 (56%) had a structured penA history documented in the electronic prescribing system (PADL note). The mean proportion of penA patients prescribed antibiotic with a PADL note ranged between 36% – 65% between June 2024 and December 2024. Excluding the run-in month and the transition month (June and December respectively).

Percentage of penA patients on any antibiotic and with a PADL note in the electronic prescribing system

|  |  |
| --- | --- |
| June | 35.7895 |
| July | 60.4762 |
| August | 53.3333 |
| September | 65.4971 |
| October | 64.5161 |
| November | 56.7416 |
| December | 53.3708 |

B3. Percentage of penA patients on any antibiotic and with a structured penA history (PADL note) in the electronic prescribing system.

Graph shows which helathcare worker de-labelled the patient

|  |  |
| --- | --- |
| AMS-P | 126 |
| CT-D | 31 |
| CT-P | 24 |
| AMS-MM | 4 |
| CT-MO | 1 |
| AMS-tech | 0 |

Figure B4. Shows which healthcare workers de-labelled patients. AMS-P antimicrobial stewardship pharmacist, CT-P clinic team pharmacist, CT-MO clinical team meds op pharm tech, CT-D clinical team doctor, AMS-tech antimicrobial stewardship technician.

Number of patients de-labelled by specialty

|  |  |
| --- | --- |
| eldercare | 32 |
| Gen surg | 30 |
| Acute med | 28 |
| resp | 15 |
| T&O | 12 |
| Renal | 10 |
| endo | 9 |
| ED | 8 |
| gastro | 8 |
| urology | 7 |
| cardiol | 5 |
| vascular | 5 |
| oncology | 4 |
| gynae | 3 |
| Ukn | 3 |
| Max fax | 2 |
| anaesthetics | 1 |
| Critical care | 1 |
| ENT | 1 |
| haem | 1 |
| neuroloy | 1 |

B5. Number of patients de-labelled in each specialty

| Specialty patient de-labelled | Number de-labelled | (%) | Number DOC | Number DDL |
| --- | --- | --- | --- | --- |
| Acute medicine | 28 | 14.97 | 6 | 22 |
| Anaesthetics | 1 | 0.53 | 0 | 1 |
| Critical care | 1 | 0.53 | 0 | 1 |
| Cardiology | 5 | 2.67 | 2 | 3 |
| Emergency Department | 8 | 4.28 | 0 | 8 |
| Eldercare | 32 | 17.11 | 10 | 22 |
| Endocrine | 9 | 4.81 | 5 | 4 |
| Ear Nose Throat | 1 | 0.53 | 1 | 0 |
| Gastroenterology | 8 | 4.28 | 6 | 2 |
| General surgery | 30 | 16.04 | 10 | 20 |
| Gynaecology | 3 | 1.60 | 0 | 3 |
| Haematology | 1 | 0.53 | 0 | 1 |
| Neurology | 1 | 0.53 | 1 | 0 |
| Oncology | 4 | 2.14 | 1 | 3 |
| Maxillo-facial surgery | 2 | 1.07 | 0 | 2 |
| Renal | 10 | 5.35 | 1 | 9 |
| Respiratory | 15 | 8.56 | 5 | 10 |
| Trauma &Orthopaedics | 12 | 6.42 | 3 | 9 |
| Unknown | 3 | 1.60 | 0 | 3 |
| Urology | 7 | 3.74 | 5 | 2 |
| Vascular surgery | 5 | 2.67 | 2 | 3 |
| total | 186 | 100 | 58 (31%) | 129 (69%) |

Table B6. Numbers of patients de-labelled in each specialty

The penicillin allergy focused history details reported by the 497 penA risk assessed patients are shown table B5. The most prevalent were; “diffuse rash or localized rash/swelling with no other symptoms “136/497 (27.4%), with index reactions occurring >10 years ago for 301/497 (60.6%), symptom onset was unknown for 254/497 (51.1%), patients weren’t hospitalised due to their reaction for 232/497 (46.7%) and patients didn’t know whether they had received treatment for their reported penA 229/497 (46.1%). Although most patients either hadn’t tolerated a penicillin since their index reaction or didn’t know (377/497 (75.9%)), 104/497 (20.9%) did have evidence of subsequent tolerance to a penicillin antibiotic, the most common were amoxicillin or amoxicillin/clavulanic acid 57/104 (54.8%).

| **Phenotype** patient grouped by AAAT phenotype.^24^ | | | **Number of patients (%)** | |
| --- | --- | --- | --- | --- |
| Diffuse rash or localized rash/swelling with no other symptoms | | | 136 (27.4) | |
| unknown reaction | | | 88 (17.7) | |
| gastrointestinal symptoms without other organ involvement | | | 42 (8.5) | |
| angioedema | | | 39 (7.8) | |
| immediate diffuse rash | | | 28 (5.6) | |
| Anaphylaxis or unexplained collapse | | | 25 (5.0) | |
| childhood exanthem | | | 21 (4.8) | |
| Pustular, blistering or desquamating rash | | | 18 (3.6) | |
| purported symptoms not on AAAT | | | 16 (3.2) | |
| urticaria | | | 16 (3.2) | |
| unknown (unable to obtain hx) | | | 16 (3.2) | |
| respiratory compromise | | | 12 (2.4) | |
| patient denies allergy | | | 12 (2.4) | |
| generalised swelling | | | 7 (1.4) | |
| laryngeal involvement | | | 6 (1.2) | |
| fever | | | 5 (1.0) | |
| Mild neurological manifestations | | | 4 (0.8) | |
| Severe liver injury, failure or DILI | | | 2 (0.4) | |
| severe neurological manifestation | | | 2 (0.4) | |
| family history of penicillin allergy only | | | 1 (0.2) | |
| mucosal ulceration | | | 1 (0.2) | |
| **Years since index reaction** | | | **Number of patients (%)** | |
| >10 years ago | | | 301 (60.6) | |
| unknown | | | 64 (12.9) | |
| 1-5 years ago | | | 43 (8.7) | |
| 6-10 years ago | | | 28 (5.6) | |
| not documented or unable to obtain history | | | 22 (4.4) | |
| <1 year | | | 19 (3.8) | |
| patient denies allergy | | | 12 (2.4) | |
| <10 years ago (recorded as such in the penA history, not de-lineated as either 1-5 or 6-10 years ago) | | | 7 (1.4) | |
| **Symptom onset** | | | **Number of patients (%)** | |
| unknown | | | 254 (51.1) | |
| <2 hour | | | 109 (21.9) | |
| >24 hours | | | 40 (8.0) | |
| 7-24 hours | | | 33 (6.6) | |
| 2-6 hours | | | 26 (5.2) | |
| unknown (unable to obtain hx) | | | 17 (3.4) | |
| unknown pt unaware of penA | | | 12 (2.4) | |
| not documented or unable to obtain | | | 5 (1.0) | |
| **Hospitalisation due to index reaction? Y/N?** | | | **Number of patients (%)** | |
| No | | | 232 (46.7%) | |
| Unknown | | | 138 (27.8%) | |
| Already an inpatient at time of reaction | | | 51 (10.3) | |
| Yes | | | 45 (9.1%) | |
| Other not documented, unable to obtain history, patient unaware of penA | | | 31 (6.2) | |
| **Treatment for the reaction?** | | | **Number of patients (%)** | |
| Unknown by patient (patient unable to recall) | | | 229 (46.1%) | |
| No treatment | | | 189 (38.0%) | |
| antihistamine | | | 16 (3.2%) | |
| unable to obtain history | | | 16 (3.2%) | |
| Unknown, patient unaware of penA record | | | 12 (2.4%) | |
| topical treatment | | | 10 (2.0%) | |
| Something but unknown what was given | | | 8 (1.6%) | |
| adrenaline | | | 6 (1.2%) | |
| unknown (not documented) | | | 4 (0.8%) | |
| injections unknown | | | 3 (0.6%) | |
| steroids | | | 2 (0.4%) | |
| unknown (not documented) | | | 1 (0.2%) | |
| other (oxygen) | | | 1 (0.2%) | |
| **Tolerated a penicillin antibiotic since index reaction?** | | | | **No. of patients (%)** |
| Yes |  | Name of penicillin | | 104 (20.9) |
|  | Amoxicillin | 31 | |  |
|  | Amoxicillin plus at least one other penicillin | 20 | |  |
|  | Flucloxacillin | 19 | |  |
|  | Flucloxacillin plus at least one other penicillin | 4 | |  |
|  | co-amoxiclav | 6 | |  |
|  | penicillin (s) unspecified | 9 | |  |
|  | Piperacillin-tazobactam | 12 | |  |
|  | pivmecillinam | 2 | |  |
| No | | | | 283 (56.9) |
| Unable to obtain hx | | | | 16 (3.2) |
| unknown | | | | 94 (18.9) |

Table B7. Penicillin allergy focused histories from the 497 patients.

**B8; reasons for declining PADL**

Sixty patients declined PADL; 29 (48.3%) didn’t provide a reason, 10 (16.1%) reported themselves too unwell, 7 (11.3%) lacked capacity, 11 (17.7%) had concerns about having a reaction, 1 (1.6%) patient not willing to consent to oral challenge instead wanting an intravenous challenge, 2 (3.2%) patients were concerned it would delay their discharge from hospital and 1 (1.6%) was absent from their bed space and therefore unable to consent. Eight patients consented to PADL but permission to DOC from the consultant responsible for the patient was not provided for the following reasons: frailty/poor prognosis (6 patients), unable to contact consultant (2 patients).

A further 18 low risk patients were excluded for clinical reason: clinical instability with an oxygen requirement, haemodynamic instability or end of life 10 (55.6%) patients, oral route unavailable 4 (22.2%) patients, imminent discharge 2 (11.1%) patients and high dose glucocorticosteroids, 2 (11.1%) patients.

|  | number of patients | Evidence of communication from secondary care to remove penA record from GP records (in discharge letter 51, dedicated PADL letter 1) | No evidence of communication from secondary care to remove penA record from GP records |
| --- | --- | --- | --- |
| Total not requiring GP communications* | 44 | 18 | 26 |
| Nil penA on SCR prior to PADL | 43 | 17 | 26 |
| Unable to view SCR | 1 | 1 |  |
|  |  |  |  |
| Total requiring GP communication because penA on SCR | 138 | 94 (68.1%) | 42 (30.4%) |
| No change to SCR | 92 (66.7%) | 51 (54.3%) | 40 (95.2%) |
| Yes, SCR updated to reflect new penA status | 45 (32.6%) | 42 (44.7%) | 2 (4.8%) |
| No SCR | 1 | 1 |  |

Table B9. Evidence of communications with the GP surgery from the hospital about de-labelled patients new penA status and whether the GP records were updated, or not.

**Appendix C Economic evaluation see Excel sheet**
